# Graft Biology in the CRISPR Era: From Tissue Fusion to Genome Compatibility

**DOI:** 10.1002/pei3.70208

**Published:** 2026-09-18

**Authors:** Melagani Subash Chandra Gowda Sahaja, Melagani Subash Chandra Gowda Sahana, Revathi Thottathil, Chithra Rekha, Poomangalath Priyada, Vidhu Sankar Babu

**Affiliations:** ^1^ Department of Plant Sciences Manipal School of Life Sciences, Academy of Higher Education Manipal Karnataka India

**Keywords:** autotetraploidy, CRISPR/Cas genome editing, developmental plasticity, embryonic grafting, graft compatibility, vascular reconnection

## Abstract

Plant lineage has traditionally constrained grafting compatibility, with monocots generally considered incompatible because of their dispersed vascular bundles and limited secondary growth. Recent studies have shown that embryonic grafting can establish successful graft unions in selected monocot systems by exploiting early developmental plasticity before anatomical constraints become fully established. Experimental evidence from cereals and orchids has demonstrated callus adhesion, vascular reconnection, and early tissue integration under controlled conditions, indicating that embryonic grafting represents a promising developmental approach distinct from conventional grafting. However, successful graft union formation does not necessarily ensure long‐term functional integration. Current evidence indicates that distant grafts may exhibit developmental desynchronization, endoplasmic reticulum stress‐associated reproductive defects, genomic dosage imbalance, and sterility, while reproducibility across taxa and translation beyond controlled environments remains poorly understood. Genome‐editing studies have identified sterility‐associated genes (ORF3/4/5, Ms1, and S‐RNase), flowering regulators (Hd1 and FT homologs), and meristem‐vascular identity genes (WUS, CUC/LOB, and MADS‐box family members) as candidate targets for investigating mechanisms underlying compatibility and reproductive stability. However, the application of these molecular approaches to embryonic graft‐derived systems remains largely unexplored. This review synthesizes current advances in embryonic grafting, discusses the molecular basis of graft compatibility and reproductive stability, and highlights key challenges and future research directions for integrating developmental biology with genome editing to improve graft success across wider taxonomic boundaries.

## 1 Introduction

For decades, graft compatibility among plant species has largely been constrained to taxonomic boundaries, typically restricting successful grafts to closely related species or genera (Muzik and La Rue 1954; Mudge et al. 2009; Melnyk 2017). Attempts to combine the unique physiological, developmental, and ecological traits of multiple plant species into a single organism have been limited (Augstein and Melnyk 2025). However, increasing challenges associated with soil health, climate change, and crop diversity have stimulated research to understand and, where possible, overcome these naturally occurring barriers.

The advanced technique of embryonic‐stage grafting has recently emerged as a promising experimental approach for investigating graft compatibility beyond conventional taxonomic boundaries (Reeves et al. 2022). Experimental studies in selected monocot systems have demonstrated successful graft union formation, early vascular reconnection, and tissue integration under controlled conditions, suggesting that embryonic tissues possess greater developmental plasticity than mature organs. This approach may facilitate the exchange of beneficial physiological characteristics, including tolerance to stress, resistance to disease, and adaptive growth potential (Jin et al. 2025). However, its broader applicability across diverse taxa and its long‐term functional stability remain to be established.

Cross‐taxon grafting, as an emerging experimental approach in developmental biology, provides a valuable system to investigate vascular compatibility, long‐distance signaling, and cellular plasticity (Thomas et al. 2022). It may also complement traditional genetic engineering approaches in species with recalcitrant transformation protocols or where genetically modified crops face strict regulatory constraints (Rubab et al. 2025). Although overcoming phylogenetic grafting barriers remains a significant scientific challenge, continued advances in developmental biology, grafting technologies, and molecular genetics may contribute to expanding graft compatibility for sustainable crop improvement.

BOX 1 **Key Determinants of Successful Graft Union Formation.**

Based on current evidence from graft biology, successful graft union formation is generally associated with the following factors:

- Cell–cell adhesion: Facilitated by callus formation and cell wall remodeling processes, including the activity of pectin‐modifying enzymes.
- Vascular reconnection: Alignment and integration of xylem and phloem between partners.
- Hormonal signaling: Auxin, cytokinin, ethylene, and other phytohormones coordinate wound healing, cell division, vascular differentiation, and graft union establishment.
- Developmental synchronization: Similar developmental stages and coordinated growth between graft partners are generally associated with improved graft union formation.

Box 1 Key biological determinants associated with successful graft union formation, summarized from current graft biology literature (Melnyk 2017; Thomas et al. 2022; Feng et al. 2024; Kragler and Bock 2025).

### 1.1 Conventional Grafting: Compatibility, Limitations, and Cross‐Taxon Barriers

Plant grafting has been widely used for centuries to improve vigor, combine favorable traits, and develop elite cultivars by integrating desirable characteristics of genetically distinct scion and rootstock partners (Feng et al. 2024; Augstein and Melnyk 2025; Rubab et al. 2025). It has been particularly successful in dicotyledonous species, which possess a vascular cambium, a meristematic tissue that facilitates scion‐rootstock fusion through callus formation and vascular reconnection (Jin et al. 2025; Melnyk 2017; Moore 1984). Consequently, dicots, particularly fruit trees, vegetable crops (Lee 1994; Lewis and Alexander 2008), and ornamental plants, have become the principal candidates for conventional grafting (Navarro et al. 1975; Zhang et al. 2024; Rubab et al. 2025). Beyond improving graft compatibility, conventional grafting has been extensively employed to enhance plant performance under both abiotic and biotic stresses. Numerous studies have demonstrated that grafting improves water‐use efficiency, nutrient uptake, nitrogen‐use efficiency, ion homeostasis, fruit quality, and tolerance to salinity, drought, and high‐temperature stress in horticultural crops (Rivero et al. 2003a, 2003b). These physiological improvements are primarily attributed to vigorous root systems, improved hydraulic conductivity, selective ion exclusion, and enhanced communication between scion and rootstock, thereby increasing crop productivity under adverse environmental conditions (Fernández‐García et al. 2002; Colla, Rouphael, Cardarelli, and Rea 2006; Colla, Rouphael, Cardarelli, Massa, et al. 2006; Colla et al. 2010; Edelstein et al. 2011; Nawaz et al. 2016; Singh et al. 2020; Yang et al. 2022).

The widespread adoption of grafting across vegetable, fruit, ornamental, and forest tree species further demonstrates its versatility as a sustainable crop management strategy. Comprehensive reviews have highlighted its expanding applications in improving productivity, stress tolerance, disease resistance, and resource‐use efficiency across diverse agricultural systems, while also emphasizing graft incompatibility as one of the major biological constraints limiting broader implementation (Belmonte‐Ureña et al. 2020; Pérez‐Luna et al. 2020; Darikova et al. 2011).

In contrast, monocot species lack a vascular cambium and possess distinct anatomical and developmental characteristics, making them generally unsuitable for conventional grafting (Muzik and La Rue 1954). Likewise, grafts between distantly related genera or plant families frequently fail due to poor callus formation, disrupted vascular reconnection, and developmental or physiological incompatibility (Turnbull et al. 2002; Thomas et al. 2022). These biological constraints have historically restricted grafting to closely related taxa and limited the transfer of desirable traits across wider phylogenetic boundaries (Augstein and Melnyk 2025). The principal differences between classical and embryonic grafting are summarized in Table 1.

**TABLE 1:** Comparison of conventional and embryonic grafting, highlighting how differences in developmental stage influence graft compatibility, tissue regeneration, and the potential to investigate graft formation across wider taxonomic boundaries.

Feature	Classical (Traditional) grafting	Embryonic grafting
Typical Host Plants	Mostly dicots (e.g., tomato, citrus, apple)	Dicots and monocots (e.g., rice, maize, sorghum, orchid)
Developmental Stage	Mature shoot/root tissues	Early‐stage embryonic tissues (pre‐cambial or meristematic)
Compatibility Requirements	Close genetic relationship (same genus or species)	Cross‐taxon potential (even intergeneric or interfamilial)
Anatomical Limitation	Requires vascular cambium for callus fusion	Bypasses cambium; uses plasticity of embryonic tissues
Tissue Rigidity	Lignified, less flexible	Soft, undifferentiated, high regeneration potential
Success in Monocots	Rare or unsuccessful due to structural barriers	Demonstrated success in cereals, grasses, and orchids
Integration Mechanism	Callus formation + vascular reconnection	Dedifferentiation + tissue fusion + de novo organogenesis
Molecular Interference	More immunogenic; identity markers expressed	Low immunogenicity; weak identity commitment at the early stage
Trait Transfer	Root‐shoot physiological integration only	Functional grafts with combined traits from the rootstock and scion
Application Scope	Horticulture, tree propagation	Synthetic crop design, developmental biology, cross‐taxon engineering
Regulatory Status	Non‐GMO, generally accepted	Also, non‐GMOs are more accepted than transgenic hybrids

*Note:* The table is based on current evidence from graft biology and embryonic grafting studies (Melnyk 2017; Reeves et al. 2022; Feng et al. 2024; Augstein and Melnyk 2025).

Recent experimental studies have demonstrated that embryonic‐stage grafting can establish successful graft unions in selected monocot systems under controlled conditions (Reeves et al. 2022). These findings suggest that the greater developmental plasticity of embryonic tissues may partially overcome some of the anatomical constraints associated with conventional grafting. However, the reproducibility, long‐term functional stability, and broader applicability of embryonic grafting across diverse taxa remain to be established (Bhatnagar and Dantu 2015). Consequently, embryonic grafting represents a promising experimental platform for investigating graft compatibility beyond conventional taxonomic boundaries and for laying the foundation for future studies on cross‐taxon grafting and developmental integration.

The evolution of grafting: From classical to embryonic approaches.

## 2 Embryonic Grafting: Developmental Foundations and Non‐Transgenic Promise

As an exciting new advancement in plant engineering, embryonic grafting has emerged as a promising experimental approach for investigating the integration of important physiological and developmental characteristics across taxonomically distant species without introducing foreign DNA. Reeves et al. (2022) demonstrated successful embryonic graft union formation, vascular reconnection, and long‐distance physiological communication in selected monocot systems under controlled in vitro conditions. Combining embryonic tissues in early stages can produce grafts that retain the independent genomic identities of the scion and rootstock while establishing functional physiological connections in experimentally demonstrated systems. However, the long‐term stability of these grafts across diverse taxa remains under investigation. This route of non‐transgenic grafting is particularly useful for crops that are resistant to transformation or agricultural regions governed by restrictive GMO regulations, as it may provide a complementary developmental alternative to transgenic and gene‐editing processes. Micrografting studies in *Arabidopsis* and related experimental systems established many of the methodological principles that underpin modern embryonic grafting, including precise tissue alignment, vascular reconnection, and the investigation of long‐distance signaling across graft junctions (Turnbull et al. 2002; Li et al. 2019).

The unique developmental plasticity of embryonic cells, particularly those that are meristematically reprogrammable and responsive to positional and hormonal cues, plays a key role in embryonic grafting. Unlike classical mature‐tissue grafts, which face rigid anatomical and developmental barriers, embryonic tissues have not yet developed fully differentiated vascular structures or strong tissue identity, which may facilitate tissue adhesion and vascular reconnection (Reeves et al. 2022; Melnyk 2017). This biological window may partially overcome traditional compatibility constraints, especially in monocotyledons that lack a vascular cambium and have historically been considered unsuitable for conventional grafting.

Recent advances have experimentally demonstrated successful early‐stage embryonic graft union formation in diverse monocot systems, including *Oryza sativa × Zea mays*, *Oryza sativa × Triticum aestivum*, *Oryza sativa × Sorghum bicolor*, and *Zea mays × Dendrobium ovatum* (Reeves et al. 2022; Mukundan et al. 2024). Reeves et al. (2022) further confirmed functional vascular connectivity using carboxyfluorescein diacetate (CFDA) transport assays and demonstrated long‐distance physiological communication through strigolactone complementation and disease‐resistance transfer experiments. Earlier.

Mukundan et al. (2024) reported more than 80% survival of maize‐orchid embryonic grafts under optimized in vitro conditions, with histological evidence of vascular continuity. Studies of conventional graft healing have shown that auxin regulates vascular differentiation and reconnection, whereas cytokinin promotes cell division and callus proliferation. Ethylene and jasmonic acid also contribute to wound signaling and graft establishment (Melnyk 2017; Thomas et al. 2022; Feng et al. 2024). However, the specific roles of these hormonal pathways during embryonic grafting remain to be experimentally determined.

Within embryonic grafting, success metrics go beyond initial adhesion to include long‐term physiological harmony, encompassing hormonal exchange, metabolic coordination, and reproductive development. However, embryo preparation, tissue polarity, scaffold support material, and growth regulator gradients all influence survival outcomes (Reeves et al. 2022; Mukundan et al. 2024). Although successful graft establishment has been demonstrated experimentally, reproducibility across diverse taxa and long‐term physiological stability remain important challenges. This suggests that embryonic grafting may complement molecular breeding or CRISPR‐based genome engineering rather than replace these approaches.

By combining embryonic grafting with emerging molecular tools, such as CRISPR‐mediated genome editing, QTL‐guided compatibility mapping, and induced polyploidization, future studies may identify the genetic mechanisms underlying graft compatibility and reproductive stability. However, direct experimental evidence demonstrating that these approaches improve embryonic graft compatibility is currently lacking, and their integration should therefore be regarded as an important future research direction rather than an established strategy.

### 2.1 Challenges and Limitations of Embryonic Grafting

Despite its promise, embryonic grafting presents several biological and technical challenges. This method requires the delicate handling of embryonic tissues in sterile, tightly controlled in vitro conditions. As a result, graft success is highly dependent on precise embryo manipulation, tissue alignment, and culture conditions, and survival rates may vary considerably among species combinations and experimental protocols. Although Reeves et al. (2022) and Mukundan et al. (2024) demonstrated successful embryonic graft union formation in selected monocot systems, the reproducibility of these protocols across a broader range of taxa remains largely untested.

Species‐specific developmental constraints also limit its applicability, and some tightly attached or rapidly developing embryos cannot be isolated effectively. Differences in embryo size, developmental stage, growth rate, and tissue organization may further influence graft compatibility and vascular reconnection, reducing the success of certain interspecific or intergeneric combinations. On top of that, grafted plants may lack long‐term physiological stability in terms of hormonal balance, systemic coordination, and reproductive performance. Current evidence for fertility, seed production, and transgenerational stability of embryonic graft‐derived plants remains limited, particularly in wide taxonomic combinations.

More importantly, variation in protocols and the absence of standardized methodologies across species can weaken reproducibility and scalability. Most embryonic grafting studies have been conducted under controlled laboratory conditions, and the translation of these protocols to greenhouse or field environments has not yet been systematically evaluated. Consequently, the practical scalability and agricultural applicability of embryonic grafting require further investigation before the technique can be broadly implemented for crop improvement.

### 2.2 Strategies to Overcome the Limitations

To address these limitations, various technical and biological innovations have emerged in recent years. For instance, the incorporation of microfluidic platforms and high‐resolution imaging systems may improve the precision of embryo microdissection, orientation, and alignment during graft assembly. In addition, the use of biocompatible scaffolds, including cellulose films and plant‐derived hydrogels, has been proposed to provide mechanical support at the graft junction and facilitate tissue contact during early graft establishment (Reeves et al. 2022). Although these approaches are promising, their effectiveness across different taxonomic combinations and their contribution to long‐term graft stability requires further experimental validation.

On a biochemical level, phytohormones play central roles in regulating graft union formation and vascular regeneration. Studies of conventional graft healing have demonstrated that auxin is the principal regulator of vascular differentiation, polarity establishment, and xylem‐phloem reconnection, whereas cytokinin promotes cell division, callus proliferation, and vascular tissue regeneration (Melnyk 2017; Nanda and Melnyk 2018; Thomas et al. 2022; Vanneste et al. 2025). Ethylene contributes to wound signaling and tissue repair, while jasmonic acid regulates early wound responses during graft establishment. Additional hormones, including gibberellins, abscisic acid, brassinosteroids, and strigolactones, have also been implicated in graft healing and long‐distance communication, although their precise regulatory functions remain less well understood (Melnyk 2017; Nanda and Melnyk 2018; Roy et al. 2025).

Experimental studies in conventional grafting have further shown that exogenous application of auxins and cytokinins can enhance callus formation, vascular regeneration, and graft healing. However, the optimal hormone regimes vary among species and experimental systems, and no universally standardized protocol has been established. Importantly, comparable studies evaluating exogenous hormone treatments during embryonic grafting have not yet been reported. Therefore, whether similar hormonal regulation can improve embryonic graft union formation, survival, or long‐term compatibility remains an important research question requiring experimental investigation.

Similarly, enzymatic incubation with pectinase or cellulase has been used to reduce cell wall rigidity by softening cell walls, thereby facilitating cell‐to‐cell adhesion. Standardizing embryo developmental stage, graft orientation, culture medium composition, and environmental conditions may further improve reproducibility across species (Bhatnagar and Dantu 2015; Melnyk 2017). Nevertheless, standardized embryonic grafting protocols remain unavailable for most crop species, contributing to variability in graft success among laboratories.

**FIGURE 1:**
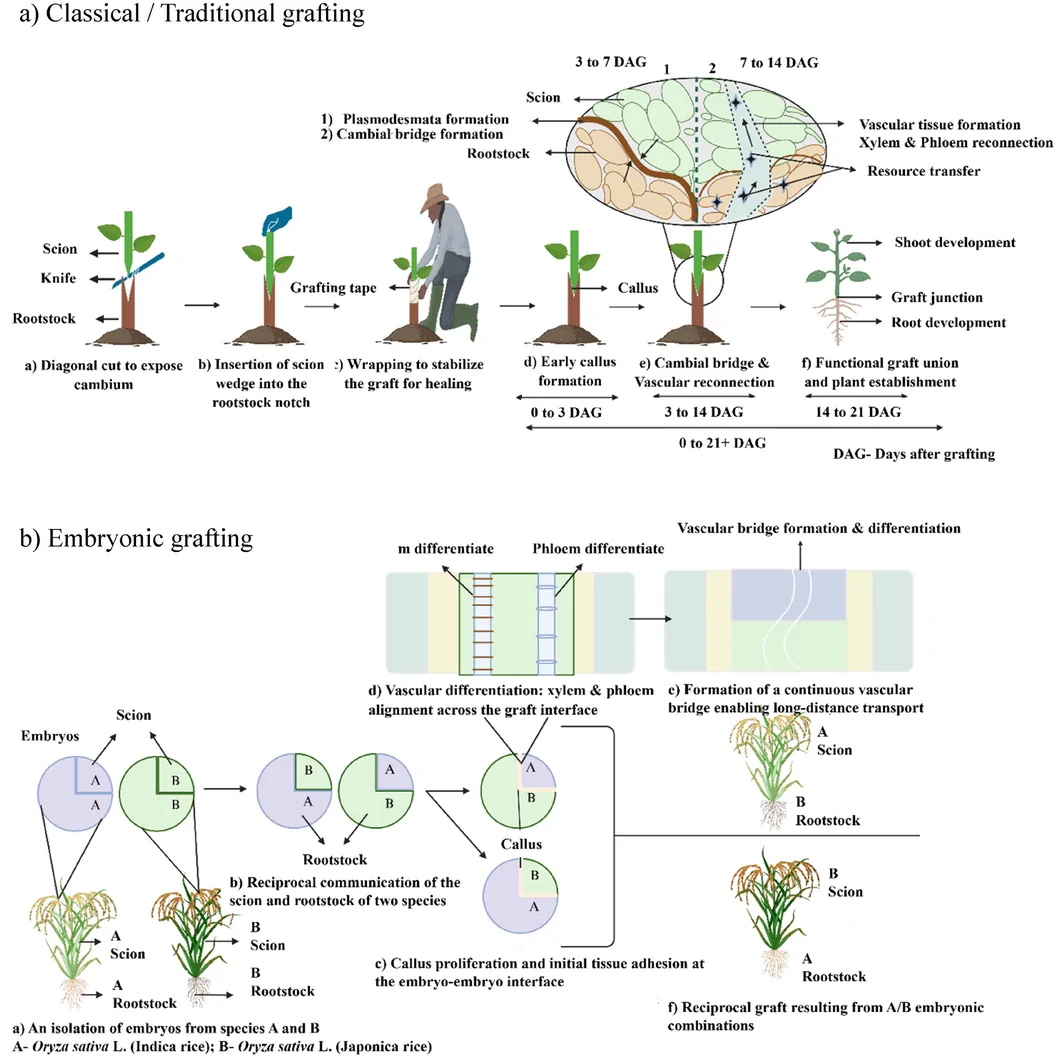
Comparative developmental trajectories of classical and embryonic grafting: From tissue fusion to functional graft formation. The figure compares the developmental sequence of conventional grafting using mature scion and rootstock tissues with embryonic grafting performed at the embryonic root–shoot interface. 3. Functional Integration Following Graft Union Formation (Melnyk 2017; Rasool et al. 2020; Reeves et al. 2022; Thomas et al. 2022; Feng et al. 2024; Kragler and Bock 2025).

Finally, embryonic grafting, tissue culture, and micropropagation strategies could be combined to further scale up propagation and facilitate downstream molecular analyses. Similarly, advances in transcriptomics, metabolomics, high‐resolution imaging, and genome editing provide valuable opportunities to investigate the molecular mechanisms underlying graft compatibility. However, their direct application to improve embryonic grafting remains largely unexplored and should currently be regarded as a future research direction rather than an established strategy (Figure 1).

Overall, these developments indicate that embryonic grafting has the potential to become a valuable platform for developmental biology and future crop improvement. However, improvements in reproducibility, scalability, survival across diverse taxa, and long‐term field performance will be essential before broader agricultural applications can be realized.

The principal biological processes contributing to successful graft union formation, including cell adhesion, vascular reconnection, phytohormone signaling, and developmental synchrony, are summarized in Box 1.

### 2.3 Physiological Harmony or Developmental Chaos?

Success in embryonic grafting marks the commencement of a new phase in which the physiological interplay between the genetically distinct scion and rootstock determines whether the resulting graft develops into a functionally integrated system or exhibits developmental instability. Following successful anatomical union, continued graft performance depends on the establishment of coordinated transport systems, hormonal signaling, and metabolic interactions between the graft partners (Reeves et al. 2022; Thomas et al. 2022).

Vascular reconnection restores xylem and phloem continuity and is essential for the transport of water, nutrients, phytohormones, and signaling molecules between the rootstock and scion. Mukundan et al. (2024) reported more than 80% survival of in vitro embryonic grafts between *Zea mays* and *Dendrobium ovatum*, with anatomical evidence of vascular continuity observed within 4–6 days after grafting. These findings demonstrate that embryonic grafting can establish rapid vascular integration under controlled laboratory conditions, although comparable studies across additional taxonomic groups remain limited.

This structural integration is accompanied by molecular reprogramming. Transcriptomic analyses of graft healing in conventional grafting systems have shown the differential expression of genes associated with meristem activity, hormone biosynthesis, cell wall remodeling, and vascular differentiation during graft union formation (Feng et al. 2024; Thomas et al. 2022). Auxin‐ and cytokinin‐mediated signaling pathways play central roles in polarity establishment, vascular regeneration, and source–sink communication during graft healing (Melnyk 2017; Kumari et al. 2023). However, comparable transcriptomic studies specifically investigating embryonic grafting remain limited.

In monocots, which traditionally lack a vascular cambium, the mesocotyl region of embryonic tissues serves as a regenerative interface that enables successful graft establishment across taxonomically distinct monocot species. Reeves et al. (2022) demonstrated successful embryonic grafts among several cereal and grass species, including rice, wheat, oats, maize, banana, and pineapple. Functional vascular continuity was confirmed using carboxyfluorescein diacetate (CFDA) transport assays, while strigolactone complementation and disease‐resistance transfer experiments demonstrated long‐distance physiological communication across the graft junction. These findings provide experimental evidence that embryonic grafting can overcome anatomical constraints previously considered to limit grafting in monocots.

Comparative studies further indicate that the developmental stage is an important determinant of graft success (Bhatnagar and Dantu 2015; Melnyk 2017). Juvenile tissues generally exhibit greater regenerative capacity, enhanced cellular plasticity, and improved vascular regeneration than mature tissues, whereas adult explants often show reduced graft success because of increased tissue differentiation and lower regenerative potential.

Mukundan et al. (2024) further observed that the *Zea mays–Dendrobium ovatum* grafts developed distinctive morphological characteristics and maintained good survival under reduced irrigation conditions. Although these observations suggest that grafting may facilitate functional interactions between physiologically distinct species, the mechanisms underlying these responses and their long‐term stability require further investigation.

Together, these studies demonstrate that embryonic grafts can establish anatomical continuity and physiological communication under controlled experimental conditions. However, evidence for long‐term reproductive stability, multigenerational performance, and field applicability remains limited. Future studies integrating hormone profiling, vascular imaging, metabolomics, and transcriptomics will be important for understanding the molecular mechanisms governing long‐term graft compatibility.

In contrast to embryonic grafting, intergeneric somatic hybridization through protoplast fusion combines the nuclear and cytoplasmic genomes of two species within a single cell. Jin et al. (2025) reported successful intergeneric somatic hybrids with improved disease resistance and abiotic stress tolerance. However, these hybrids frequently exhibit challenges including low plant regeneration efficiency, chromosome instability, irregular meiosis, and reduced fertility, particularly following wide hybridization.

Unlike somatic hybridization, embryonic grafting preserves the independent genomes of the scion and rootstock while enabling physiological communication through the graft union. This characteristic may provide a useful experimental platform for investigating cross‐species physiological interactions without altering genome composition. Nevertheless, additional experimental studies are required to determine whether embryonic grafting can be translated into stable, reproducible, and agronomically relevant crop improvement strategies across diverse plant taxa (Table 2).

**TABLE 2:** Comparison of taxonomic compatibility achieved through embryonic grafting and conventional grafting across different taxonomic levels, including intraspecific, interspecific, intergeneric, and interfamily combinations.

Taxonomic relationship	Embryonic grafting	Conventional grafting	References
Intraspecific	Wheat × Wheat; Rice × Rice	Tomato × Tomato; Eggplant × Eggplant; Pepper × Pepper	Reeves et al. (2022) and Kawaguchi et al. (2008)
Interspecific	Wheat ( *Triticum aestivum* ) × Rye ( *Secale cereale* ); Barley ( *Hordeum vulgare* ) × Wheat	Tomato ( *Solanum lycopersicum* ) × Eggplant ( *Solanum melongena* )	Reeves et al. (2022) and Kawaguchi et al. (2008).
Intergeneric	Not yet reported	Tomato ( *Solanum lycopersicum* ) × Pepper ( *Capsicum annuum* )	Kawaguchi et al. (2008)
Interfamily	Not yet reported	*Nicotiana benthamiana* × *Arabidopsis thaliana; Nicotiana benthamiana* × *Chrysanthemum morifolium*	Notaguchi et al. (2020)

*Note:* Representative plant combinations for each category are summarized (Melnyk 2017; Rasool et al. 2020; Reeves et al. 2022; Thomas et al. 2022; Feng et al. 2024; Kragler and Bock 2025).

## 3 Bridge From Developmental Promise to Molecular Barriers

While embryonic grafting opens remarkable opportunities for cross‐taxon chimerism, overcoming anatomical contradictions, and leveraging the totipotency of early‐stage cells, it does not fully escape biological constraints (Reeves et al. 2022). As the grafted tissues grow into maturity and attempt to reconcile their species differences, incompatibilities may arise below the surface, such as reproductive sterility, morphological abnormalities, or aberrant signaling between scion and rootstock. Although successful anatomical union can be achieved, sustained functional integration depends on coordinated molecular and developmental processes. Therefore, understanding the genes, signaling pathways, and stress‐response networks that regulate graft compatibility is essential for improving the reproducibility and long‐term stability of embryonic grafts.

### 3.1 The Molecular Dialogue Behind Graft Success and Failure

Grafting success is generally associated with a highly coordinated sequence of physiological, biochemical, and molecular events that unfold from the moment two wounded tissues come into contact (Augstein and Melnyk 2025). First, the adhesion is mediated by the rapid deposition of pectins, extensins, and β‐1,4‐glucanases, which remodel cell walls to form a common extracellular matrix. Auxin accumulates above the junction, and cytokinin accumulates below, forming hormonal gradients that promote cambial division and provascular identity (Melnyk 2017; Nanda and Melnyk 2018; Feng et al. 2024). Within hours, wound‐responsive transcriptional modules, *NACs*, *DOFs*, *ERFs*, and *WOX* genes initiate cell dedifferentiation, callus expansion, and the reactivation of vascular programs, such as *PXY‐WOX4*, *VND7*, *NAC020*, and *NEN4*, allowing the establishment of functional phloem and xylem connections in several conventional grafting systems, including Arabidopsis, tomato, rice, and spruce (Melnyk 2017; Feng et al. 2024; Thomas et al. 2022).

Recent systems‐level transcriptomic analyses have shown that graft success is not determined solely by anatomical proximity but also by coordinated gene regulatory networks established at the graft junction. Thomas et al. (2022) used transcriptomic profiling and gene regulatory network analyses to demonstrate that compatible grafts activate coordinated transcriptional pathways supporting cambial activation, vascular differentiation, and hormonal integration. In contrast, incompatible grafts fail to activate these regulatory pathways. *SlWOX4*, a cambium‐associated transcription factor, was identified as an important regulator that links wound perception to vascular regeneration. In compatible grafts, *SlWOX4* interacts with a conserved regulatory module that includes *PXY*, *HD‐ZIP III*, and auxin‐responsive genes that promote sustained cambial proliferation and xylem‐phloem reconnection. Conversely, incompatible graft combinations exhibit reduced *SlWOX4* expression, impaired signaling, and disrupted cell cycle progression, resulting in poor vascular continuity and delayed graft union formation (Thomas et al. 2022). These findings indicate that coordinated molecular regulation contributes to successful graft establishment in compatible grafting systems.

Another regulatory layer involves metabolic and stress‐response reprogramming. Compatible grafts typically exhibit a transient burst of reactive oxygen species (ROS), followed by activation of antioxidant enzymes including superoxide dismutase (SOD), catalase (CAT), ascorbate peroxidase (APX), and peroxidase (POD), thereby restoring cellular redox homeostasis during graft healing (Melnyk 2017; Feng et al. 2024). In contrast, incompatible graft combinations, reported in several conventional heterografting systems, including tomato‐pepper, citrus‐pomelo, and grapevine, exhibit prolonged oxidative stress, increased phenolic accumulation, reduced antioxidant activity, and delayed callus maturation (Melnyk 2017; Feng et al. 2024). In addition, sugars, ethylene, gibberellins, jasmonates, and brassinosteroids coordinate wound signaling and tissue regeneration, collectively influencing graft compatibility and vascular reconnection (Melnyk 2017; Nanda and Melnyk 2018; Feng et al. 2024).

Besides hormonal regulation and redox homeostasis, successful graft establishment also relies on extensive long‐distance molecular communication between graft partners. Mobile RNAs, proteins, peptides, and other signaling molecules coordinate vascular regeneration, developmental reprogramming, and systemic physiological responses. Transcriptomic analyses have further revealed extensive transcriptional reprogramming across graft junctions, while recent evidence suggests that plasmid‐like extrachromosomal circular DNAs can move across compatible graft interfaces, highlighting an additional layer of molecular exchange during graft establishment (Tsutsui and Notaguchi 2017; Rasool et al. 2020; Li and Zhao 2021; Zhang et al. 2024).

Although these molecular mechanisms have been well characterized in conventional grafting systems, comparable studies in embryonic grafting remain limited. Reeves et al. (2022) demonstrated successful embryonic graft formation among monocot species, including rice, wheat, oats, maize, banana, and pineapple, with functional vascular continuity confirmed through CFDA transport assays and physiological complementation experiments. Similarly, Mukundan et al. (2024) reported rapid vascular reconnection and high survival of embryonic monocot grafts under controlled in vitro conditions. However, the regulatory networks governing long‐term compatibility, reproductive development, and physiological stability in embryonic grafts remain largely unexplored.

While these pathways explain early graft union formation, successful structural integration does not necessarily guarantee long‐term developmental or reproductive compatibility. As scion and rootstock undergo further growth, differences in genome dosage, epigenetic state, hormonal regulation, and stress signaling may potentially influence reproductive performance and physiological stability. Although these processes have not yet been experimentally characterized in plants generated through embryonic grafting, studies of hybrid sterility provide valuable molecular frameworks for identifying candidate pathways that may contribute to long‐term graft compatibility and developmental stability.

Among the best‐characterized reproductive incompatibility systems is the S5 killer‐protector complex (*ORF3‐ORF4‐ORF5*) in *Oryza sativa* , together with fertility regulators such as *Ms1* in wheat and S‐RNase‐mediated incompatibility in Solanaceae (Rao et al. 2021; Okada et al. 2019). In rice, the combined activity of ORF5^+^ and ORF4^+^ induces endoplasmic reticulum (ER) stress, leading to abortion of female gametes lacking the protective ORF3^+^ allele, which encodes an Hsp70‐class molecular chaperone responsible for protein folding and stress tolerance (Rao et al. 2021). Components of the unfolded protein response (UPR), including *BiP*, *IRE1*, and *bZIP60*, further regulate ER‐stress signaling and cellular homeostasis (Feng et al. 2024).

Although direct evidence linking these pathways to embryonic graft compatibility is currently unavailable, they represent promising molecular candidates for future investigation. If similar reproductive incompatibilities are identified in graft‐derived plants, CRISPR‐mediated manipulation of *ORF3*, *ORF4*, *ORF5*, *OsMADS3*, *OsMADS58*, and *EMF2b* may improve reproductive stability and developmental synchrony (Rao et al. 2021; Zegeye et al. 2022; Zhou et al. 2023). Likewise, targeted modulation of *BiP, IRE1, and bZIP60* may help regulate excessive UPR activation if prolonged ER stress proves detrimental to graft‐derived plants (Feng et al. 2024).

Complementary to gene editing, QTL mapping could also be utilized to identify chimeric fertility interacting loci (qCFIGs). Drawing parallels from hybrid sterility studies, QTLs such as *qSIG3.1*, *qSIG3.2*, *qSIG6.1*, and *qSIG12.1* provide insights into the genetic basis of reproductive incompatibility (Rao et al. 2021). These loci may eventually be combined with marker‐assisted selection and CRISPR‐based approaches to improve compatibility and reproductive stability in future engineered chimeras.

Advanced genome editors like SpRYCas9 and xCas9 provide greater flexibility in targeting due to relaxed PAM sequence requirements, allowing access to previously inaccessible genomic regions, especially in less‐studied or difficult‐to‐modify plant species (Zegeye et al. 2022; Zhou et al. 2023).

Simultaneously, synchronizing flowering and seed formation is critical to ensure the complete reproduction of chimeras. Photoperiod‐dependent flowering regulators *Hd1*, *Hd3a*, *RFT1*, and *Ehd1* could be fine‐tuned to match shoot‐root developmental dynamics. Genes involved in grain development, including *GW2*, *GS3*, *qSW5/GW5*, *Gn1a*, and *GIF1*, also represent promising targets for future optimisation of productivity if reproductive compatibility can be achieved (Li et al. 2016; Zegeye et al. 2022; Zhou et al. 2023).

**FIGURE 2:**
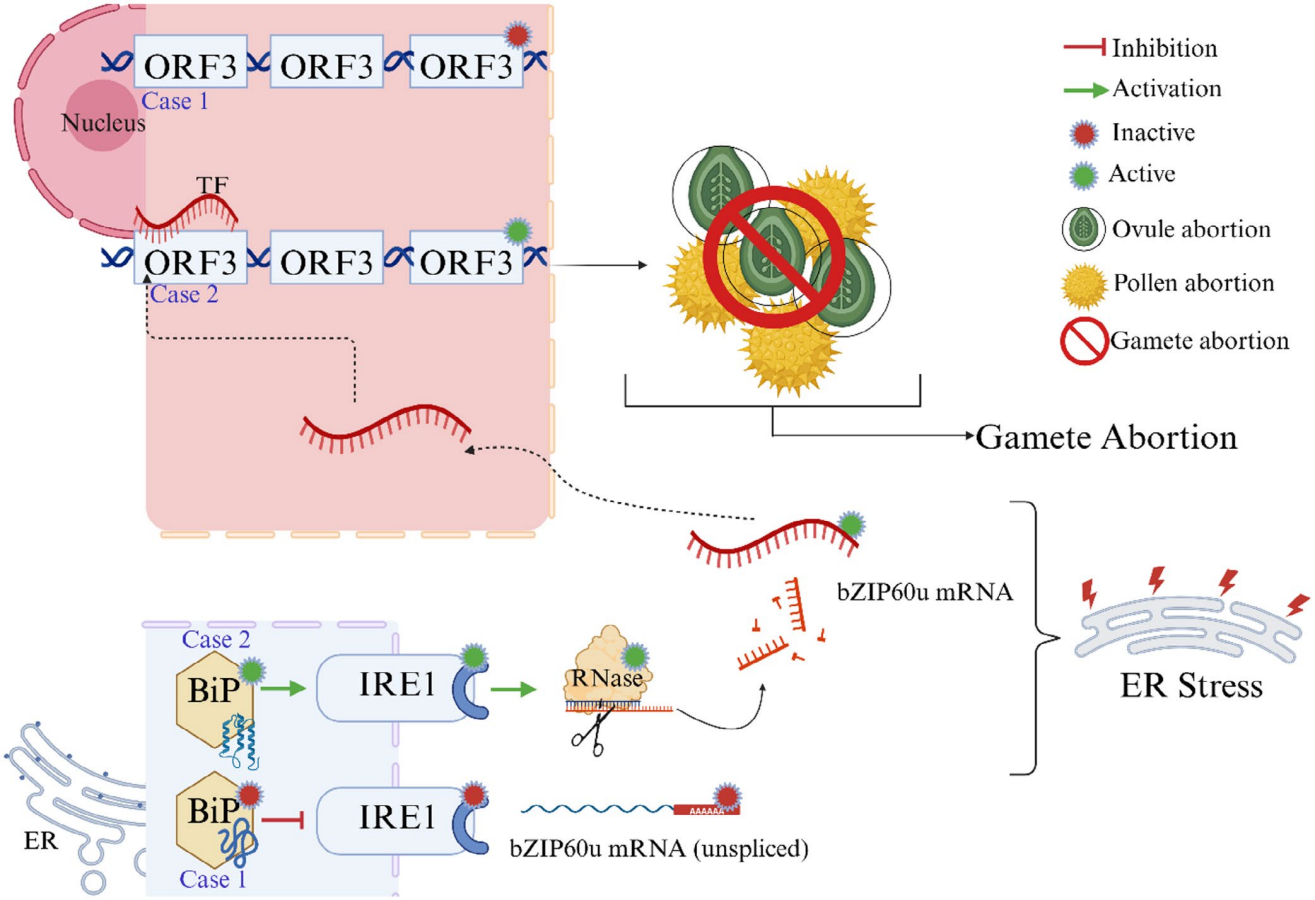
Overview of stress‐mediated reproductive breakdown in plants. This figure illustrates how activation of sterility‐associated genes (e.g., *ORF3*) can trigger endoplasmic reticulum (ER) stress, leading to reproductive failure. Under normal conditions (Case 1), *ORF* genes remain inactive, *BiP* binds to *IRE1*, and *bZIP60* mRNA remains unspliced, maintaining ER homeostasis and normal gamete development. In the stress condition (Case 2), transcriptional activation of *ORF* genes induces ER stress, causing *BiP* dissociation and *IRE1* activation. Activated *IRE1* mediates unconventional splicing of *bZIP60u* mRNA, generating a stress‐responsive transcription factor. This pathway disrupts reproductive development, resulting in pollen, ovule, or gamete abortion (Howell 2013; Rao et al. 2021).

Collectively, current evidence suggests that successful graft formation depends on coordinated regulation of cell wall remodeling, hormonal signaling, vascular regeneration, transcriptional reprogramming, and oxidative stress responses. While these mechanisms are increasingly understood in conventional grafting, future studies integrating embryonic grafting with transcriptomics, genome editing, and molecular breeding will be essential to determine whether these same regulatory networks govern long‐term compatibility in cross‐taxon embryonic grafts and to facilitate the rational design of stable, reproductively competent graft plants (Figure 2).

## 4 Targeted Trait Engineering in Cereals and Solanaceae: A Model for Chimera Optimization

Recent advances in CRISPR technology extend beyond conventional gene knockout approaches to include multiplex genome editing, RNA‐targeting systems, nanocarrier‐assisted delivery, and precision editing platforms. These emerging technologies increase the capacity to manipulate multiple developmental, reproductive, and stress‐responsive pathways simultaneously, thereby providing valuable opportunities for future engineering of graft compatibility and long‐term stability in complex plant systems (Mushtaq et al. 2018; Mujtaba et al. 2021; Gohil et al. 2024; Zhang et al. 2019).

Combined knowledge from developmental biology and genome editing has ushered in a new era in understanding compatibility, fertility, and regeneration capacity among diverse organisms. The four model crops in the context of intergeneric grafting, rice (Bin Rahman and Zhang 2023), wheat, tomato, and potato, provide complementary molecular models to understand how reproductive barriers, regeneration plasticity, and metabolic tuning have been studied experimentally.

These model crops collectively represent valuable experimental systems for identifying candidate genes and pathways that may influence graft integration and provide molecular frameworks for future CRISPR‐based studies aimed at improving embryonic graft compatibility. However, most of these studies were conducted in conventional breeding, developmental biology, or functional genomics rather than embryonic grafting.

### 4.1 Rice: A Blueprint for Reproductive Synchronization and Trait Stabilization

Rice has become the most mature monocot model for CRISPR/Cas‐mediated trait engineering. Rice genome‐editing studies have successfully identified genes controlling hybrid sterility, photoperiod sensitivity, grain architecture, yield, and stress adaptation, many of which represent important candidate pathways for future investigations into inter‐taxon graft compatibility (Bhattacharya 2011; Zegeye et al. 2022; Zhou et al. 2023).

Editing of *TMS5*, *TMS10*, and *CSA* has enabled the development of thermo‐ and photoperiod‐sensitive male‐sterile lines for efficient hybrid seed production (Zegeye et al. 2022). Hybrid sterility between *indica* and *japonica* rice has also been alleviated through editing of *SaF*, *OgTPR1*, *Sc*, and *ORF2*, restoring gamete viability and seed production (Rao et al. 2021; Zegeye et al. 2022).

CRISPR‐mediated editing of *Gn1a*, *DEP1*, *GS3*, and *IPA1* has successfully improved grain architecture and yield‐related traits by increasing panicle size, grain number, and overall productivity (Li et al. 2016). In addition, other important yield‐associated genes, including *GW2* and *TGW6*, have been identified as promising genome‐editing targets for improving grain size and grain weight (Zegeye et al. 2022; Zhou et al. 2023). Aroma and photoperiod sensitivity have likewise been enhanced through editing of *BADH2* and *Hd1*, respectively (Zegeye et al. 2022; Zhou et al. 2023).

Rice CRISPR platforms predominantly employ SpCas9‐NG, xCas9, and SpRYCas9, together with OsU6 and 2 × 35S promoters and ribonucleoprotein (RNP)‐mediated delivery systems to achieve efficient, transgene‐free genome editing (Zegeye et al. 2022; Zhou et al. 2023).

Collectively, these studies establish rice as one of the best‐characterized systems for reproductive development, flowering regulation, and stress‐response genetics. Although these genes have not yet been investigated directly in embryonic grafting, they represent promising candidate targets for future studies aimed at improving reproductive synchronization and long‐term stability of graft‐derived plants.

### 4.2 Wheat: Overcoming Polyploid Redundancy for Predictable Reproductive Control

Wheat provides a pronuclear fusion perspective that contrasts with the sterilizing gene‐dominated seed‐tracking concept. The complex hexaploid genome of wheat confounds the predictability of phenotypic output from targeted editing in discrete genomic locations, as successful trait alteration must account for the complexity underlying quantitative multi‐locus trait determination within the polyploid genome. For example, the disruption of Mendelian‐targeted genes generating male sterility as a system to reduce the costs and complexities of hybrid seed production required targeted editing of the *Ms1*, *Ms‐A1*, and *Ms‐D1* (Okada et al. 2019), encoding lipid transfer proteins essential for pollen exine development within wheat pollen grains. This RNA‐directed DNA nucleolytic approach successfully generated a targeted male sterility phenotype, providing a path for predictable hybrid wheat production achieved without laborious deployment of restorer genes. Multiplexed gRNAs afforded a specific target in all three homoeologous sequences due to conserved sequences in the first exon. This precisely targeted editing approach within a complex genome demonstrates the power and flexibility of CRISPR systems, where modifications designed and implemented for the same outcomes as classical breeding practices occur in generations rather than decades. The precise modifications introduced using Agrobacterium and biolistic systems into embryogenic calli were transmitted steadily to subsequent generations and allowed for the recovery of segregated Cas9‐free male‐sterile wheat lines (Wang et al. 1997; Okada et al. 2019). More recently, modified Cas9 variants such as SpCas9‐NG, xCas9, and SpRYCas9 have effectively expanded targeted editing in broad PAM sequence spaces in the hexaploid wheat promoter, providing prospects for sterility modules (Zhou et al. 2023).

Collectively, these studies demonstrate that CRISPR can effectively manipulate reproductive pathways even within highly complex polyploid genomes. These experimentally validated mechanisms may provide useful molecular candidates for investigating reproductive stability and genome dosage balance in embryonic graft‐derived plants. However, their application to graft compatibility remains hypothetical and requires experimental validation.

### 4.3 Tomato: Organ Identity, Vascular Regeneration, and Redundancy Resolution

Tomato from the Solanaceae provides a robust dicot model for the study of meristem identity, organ boundary formation, and vascular reconnection post‐grafting. Genome‐scale CRISPR libraries designed against the families of *MADS‐box*, *NAC*, *ARF*, and other transcription factor families revealed subtle phenotypes masked by paralogs (Berman et al. 2025). CRISPR modifications of *WUS*, *CLV3*, *LOB*, and *CUC* genes revealed control of meristem size, callus formation, and vascular continuity (Thomas et al. 2022; Tiwari et al. 2023). CRISPR‐based edits in *SlMADS3*, *SlAGL6*, and *Sl‐SEP4* show control of floral identity and modulation of sterility‐fertility. Genes responsible for pigment production (*PSY1*, *HY5*), fruit shelf life (*RIN*), and pathogen resistance (*Mlo1*, *SlDMR6*) illustrate the plasticity of metabolism (Berman et al. 2025; Thomas et al. 2022). Tomato, therefore, serves as an important experimental model for understanding developmental patterning, meristem maintenance, and vascular regeneration. These experimentally characterized pathways may guide future studies aimed at improving graft union formation and long‐term compatibility in embryonic grafting systems (Thomas et al. 2022; Feng et al. 2024).

### 4.4 Potato: Polyploid Editing and Metabolic Reprogramming in Heterozygous Genomes

Potato presents an opportunity to gain similar insights on editing highly heterozygous, polyploid genomes, as in graft systems, where nuclear‐cytoplasmic genome interactions significantly reduce graft union compatibility and maintenance (Tiwari et al. 2022). Editing *GBSSI*, *SBE1/2*, and *PPO2* reprogrammed starch biosynthesis and reduced enzymatic browning, respectively, which are plasticity strategies for metabolic rewiring that likely parallel nutrient transport and assimilation in chimeras. CRISPR‐mediated edits of *eIF4E1*, *StDMR6*, and *StCHL1* generated a broad spectrum of pathogen resistance (Tiwari et al. 2022; Chincinska et al. 2023). S‐RNase editing in a diploid form of potato produced self‐compatible lines, which might provide novel conceptual parallels to breaking sterility in inter‐taxon systems. Allele‐specific, base editing, and Cas12a‐mediated multiplexing strategies overcome genome redundancy and mosaicism. Beyond genome editing, heterografting studies between tomato and potato have demonstrated coordinated physiological and transcriptomic responses across the graft junction. Comprehensive transcriptome analyses revealed that successful scion–rootstock integration involves extensive reprogramming of genes associated with photosynthesis, primary metabolism, hormone signaling, and stress responses, highlighting the molecular coordination required for stable graft establishment (Zhang and Guo 2019; Zhang et al. 2019).

Collectively, potatoes provide a useful model for studying metabolic plasticity, stress adaptation, and genome editing in highly heterozygous genomes. Whether these mechanisms contribute directly to embryonic graft compatibility remains unknown and warrants future investigation. The major CRISPR‐edited genes and their experimentally validated functions across rice, wheat, tomato, and potato are summarized in Table 3.

**TABLE 3:** Key gene modules regulating graft success, fertility, yield, and developmental synchronization across major crops.

Crop	Gene(s)	Functional category	Experimentally demonstrated role	Editing strategy	Key references
Rice ( *Oryza sativa* L.)	*ORF3, ORF4*, *ORF5*	Hybrid sterility/ER stress	Controls female gamete viability and hybrid sterility through ER‐stress signaling	CRISPR knockout/CRISPR activation	Rao et al. (2021) and Zegeye et al. (2022)
	*BiP*, *bZIP60*, *IRE1*	Unfolded Protein Response (UPR)	Regulate ER‐stress responses and protein‐folding homeostasis	CRISPR interference	Howell (2013) and Feng et al. (2024)
	*OsMADS3*, *OsMADS58*, *EMF2b*	Floral development	Stamen identity, ovule, and embryo sac development	Expression modulation	Zhou et al. (2023) and Zegeye et al. (2022)
	*Hd1*, *Hd3a*, *RFT1*, *Ehd1*	Flowering regulation	Regulate photoperiod‐dependent flowering	Base editing/CRISPRa	Zegeye et al. (2022) and Zhou et al. (2023)
	*GW2*, *GS3*, *qSW5*, *Gn1a*, *GIF1*	Grain development	Grain size, grain number, and grain filling	Knockout/multiplex editing	Li et al. (2016), Han et al. (2025) and Zhou et al. (2023)
Wheat ( *Triticum aestivum* L.)	*Ms1, Ms‐A1, Ms‐D1*	Male fertility	Tapetum development and pollen viability	Multiplex CRISPR	Okada et al. (2019)
	*Vrn1, Vrn2, Vrn3, FT1*	Flowering	Vernalization and flowering time	Promoter editing	Zhou et al. (2023)
	*TaGW2, TaCKX2.1*	Yield	Grain size and grain number	Knockout	Zhou et al. (2023)
	*TaEPFL10, TaMLO*	Stress/disease	Disease resistance and stomatal regulation	Knockout	Zhou et al. (2023)
Tomato ( *Solanum lycopersicum* L.)	*SlWOX4*	Vascular regeneration	Cambial activity, vascular regeneration, and graft union formation	CRISPR‐based functional studies	Thomas et al. (2022)
	*WUS, CLV3, CUC, LOB*	Meristem identity	Regulate meristem maintenance, organ boundaries, and regeneration	CRISPR activation/editing	Berman et al. (2025) and Tiwari et al. (2023)
	*SlMADS3, SlAGL6, SlSEP4*	Floral identity	Flower development and fertility	Multiplex CRISPR	Berman et al. (2025) and Zhou et al. (2023)
	*RIN, PSY1, HY5*	Fruit ripening/metabolism	Ripening, carotenoid synthesis, and photomorphogenesis	Editing	Berman et al. (2025) and Tiwari et al. (2023)
	*SlMLO1, SlDMR6, ARF, NAC*	Defense/hormone signaling	Disease resistance and developmental regulation	Knockout	Berman et al. (2025), Feng et al. (2024), and Tiwari et al. (2023)
Potato ( *Solanum tuberosum* L.)	*PPO2*	Browning	Enzymatic browning	Knockout	Tiwari et al. (2022)
	*GBSSI, SBE1, SBE2*	Starch metabolism	Tuber starch composition	Knockout	Tiwari et al. (2022)
	*eIF4E1, StDMR6*	Disease resistance	Virus and pathogen resistance	Knockout	Chincinska et al. (2023)
	S‐RNase	Self‐incompatibility	Self‐compatibility and fertility restoration	Knockout	Enciso‐Rodriguez et al. (2019)

**FIGURE 3:**
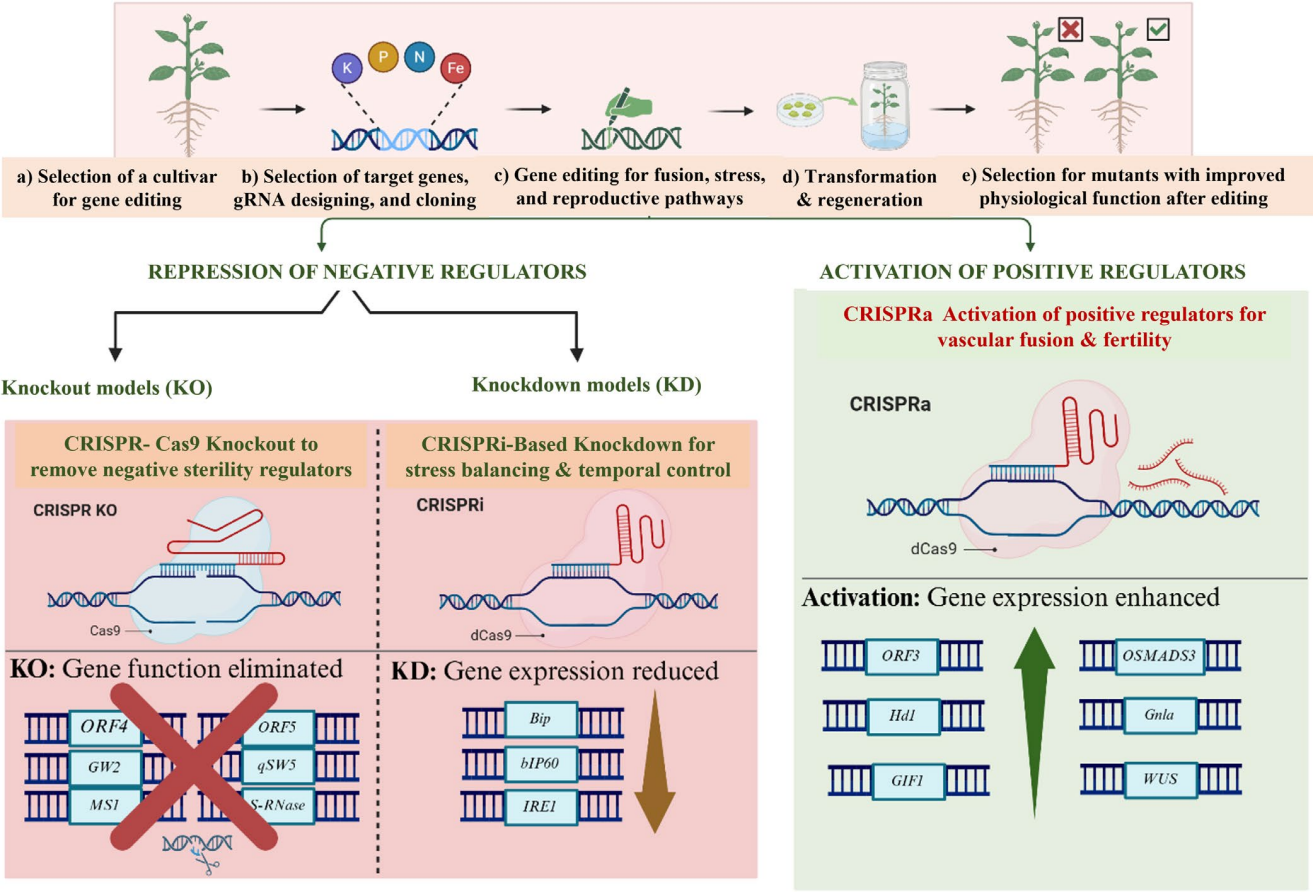
Conceptual framework illustrating potential CRISPR‐based strategies for improving graft compatibility and reproductive stability in graft‐derived chimeras. The proposed workflow includes cultivar selection, identification of candidate genes, guide RNA (gRNA) design, genome editing, and regeneration of edited plants. CRISPR/Cas9‐mediated knockout of sterility‐associated or negative regulatory genes, CRISPR interference (CRISPRi) to reduce stress‐related gene expression, and CRISPR activation (CRISPRa) to enhance positive regulators of fertility, meristem function, and vascular development are presented as potential approaches. These strategies are proposed to facilitate vascular integration, developmental coordination, and reproductive stability in future graft‐derived chimeras; however, their application in embryonic graft‐derived systems remains hypothetical and requires experimental validation (Song et al. 2015; Mujtaba et al. 2021; Tiwari et al. 2022, 2023; Chincinska et al. 2023; Zegeye et al. 2022; Feng et al. 2024; Kragler and Bock 2025).

Overall, rice, wheat, tomato, and potato provide complementary experimental systems for understanding reproductive development, regeneration, vascular differentiation, and stress adaptation. While the CRISPR studies discussed above were conducted primarily in breeding and functional genomics rather than embryonic grafting, they identify candidate genes and regulatory pathways that could be evaluated in future efforts to improve graft compatibility, developmental synchronization, and long‐term stability of cross‐taxon chimeras (Figure 3).

## 5 Integrative Perspective: Cross‐Taxon Lessons for Chimera Engineering

Advancements in rice, wheat, tomato, and potato collectively provide valuable molecular frameworks for understanding developmental, physiological, and reproductive compatibility across monocot and dicot crops. Studies in these model species have identified conserved genetic pathways regulating fertility, flowering, vascular regeneration, stress responses, and metabolic coordination, which may inform future strategies for engineering stable cross‐taxon chimeras. Rice and wheat primarily contribute insights into reproductive synchronization and gene dosage control in monocots. In contrast, tomato and potato provide complementary knowledge of vascular regeneration, meristem maintenance, and metabolic plasticity in dicots.

Although these genetic modules have not yet been experimentally integrated into embryonic grafting systems, they offer promising molecular targets for future optimization of graft‐derived chimeras. Potential applications include:

- Fertility control modules, including *TMS5/TMS10* in rice, *Ms1* in wheat, and S‐RNase in potato, could potentially be manipulated to regulate reproductive compatibility and fertility restoration in future chimeric systems.
- Developmental regulators, including MADS‐box genes, *WUS, CLV3, LOB, CUC, and SlWOX4* may contribute to polarity establishment, vascular regeneration, and graft junction stabilization.
- Metabolic and stress‐associated genes, including *GS3, PPO2, DMR6*, and genes involved in ROS homeostasis, may improve nutrient allocation, physiological coordination, and stress tolerance between scion and rootstock.
- Multiplex CRISPR genome editing could enable simultaneous modification of multiple compatibility‐associated loci in both graft partners, providing a future strategy to minimize developmental incompatibility and improve long‐term physiological integration. The candidate genetic modules discussed above, together with their mechanistic functions and potential relevance to future cross‐taxon grafting, are summarized in Table 4.

Collectively, these advances suggest that combining embryonic grafting with modern genome editing represents a promising future direction for plant chimera engineering. However, experimental validation will be required to determine whether these molecular interventions can improve graft compatibility, reproductive stability, and long‐term performance in embryonic graft‐derived chimeras.

**TABLE 4:** CRISPR‐editable gene modules associated with fertility regulation, developmental coordination, vascular regeneration, and physiological stability in model crop species.

Module	Representative genes	Mechanistic function	Potential relevance to cross‐taxon grafting	Key references
ER stress and sterility control	*BiP, bZIP60, IRE1, ORF3, ORF4, ORF5*	Unfolded Protein Response (UPR), ER‐stress signaling, gamete protection	Potential regulation of graft‐induced reproductive incompatibility and sterility	Melnyk (2017), Rao et al. (2021), Feng et al. (2024) and Howell (2013)
Floral transition and synchrony	*Hd1, Hd3a, RFT1, Ehd1, FT1, Vrn1, Vrn2, Vrn3*	Photoperiod sensing and flowering regulation	Synchronization of reproductive development between graft partners	Zegeye et al. (2022) and Zhou et al. (2023)
Organ identity and floral development	*OsMADS3, OsMADS58, SlMADS3, SlAGL6, EMF2b*	Floral organ specification, meristem identity, and reproductive development	Improved developmental coordination and reduced reproductive abnormalities	Zegeye et al. (2022), Zhou et al. (2023), and Berman et al. (2025)
Callus formation and vascular reconnection	*SlWOX4, WUS, CLV3, CUC, LOB, ARF, NAC*	Stem cell maintenance, cambial activation, vascular regeneration	Enhanced graft union formation and vascular integration	Thomas et al. (2022), Melnyk (2017), and Feng et al. (2024)
Yield and grain/tuber development	*GW2, GS3, qSW5/GW5, Gn1a, TaGW2, TaCKX2.1, GIF1*	Regulation of grain size, grain number, and assimilate partitioning	Improved productivity and source–sink coordination in engineered chimeras	Li et al. (2016), Wang et al. (2017), Han et al. (2025), and Zhou et al. (2023)
Plant immunity and stress resilience	*DMR6, MLO, MYB44*	Disease resistance, defense signaling, abiotic stress responses	Improved long‐term physiological stability and post‐graft resilience	Tiwari et al. (2022), Chincinska et al. (2023), and Feng et al. (2024)

*Note:* These genes represent potential molecular targets for future engineering of compatibility, reproductive performance, and long‐term stability in embryonic graft‐derived cross‐taxon plants.

In addition to molecular engineering, chromosome doubling (induced allotetraploidy) represents a potential strategy for overcoming post‐graft sterility and genome dosage imbalance in cross‐taxon chimeras. In other plant breeding systems, antimitotic agents such as colchicine and oryzalin have been widely used to induce chromosome doubling, restore homologous meiotic pairing, improve fertility, and stabilize hybrid genomes. Although this approach has not yet been investigated in embryonic graft‐derived plants, combining induced polyploidization with CRISPR‐mediated genome editing may provide a complementary strategy for improving reproductive stability and long‐term compatibility in future studies (Dhooghe et al. 2011; Zegeye et al. 2022).

## 6 Chromosome Doubling as a Genomic Rescue Strategy

Beyond molecular engineering strategies, genome‐scale approaches such as induced polyploidization have been widely employed in plant breeding to restore fertility, improve meiotic stability, and rebalance genome dosage in interspecific and intergeneric hybrids. Although chromosome doubling has not yet been experimentally investigated in embryonic graft‐derived plants, it represents a promising complementary strategy for improving reproductive stability and physiological homeostasis in future cross‐taxon grafting systems. By increasing chromosome number, induced polyploidization may reduce genomic imbalance, improve homologous chromosome pairing during meiosis, and enhance developmental coordination between genetically distinct tissues (Pawlowski 2010; Schubert and Shaw 2011). Consequently, combining chromosome doubling with genome editing offers a conceptual framework for overcoming reproductive barriers that may arise following embryonic grafting.

Mitotic chromosome doubling is typically induced using antimitotic agents that disrupt spindle fiber formation during mitosis, thereby preventing chromosome segregation and resulting in chromosome doubling. Among these compounds, colchicine, oryzalin, trifluralin, and amiprophos‐methyl (APM) are the most widely used. Colchicine is generally applied at concentrations of 1.25–2.5 mM, whereas oryzalin, trifluralin, and APM are effective at considerably lower concentrations, typically 1–50 μM, depending on the plant species, explant type, and treatment duration (Dhooghe et al. 2011). Since these antimitotic agents differ in cellular uptake, microtubule‐binding affinity, and cytotoxicity, optimal treatment conditions must be established individually for each species and explant.

The effectiveness of chromosome doubling is strongly influenced by explant type, treatment duration, and chemical concentration. Antimitotic agents may be applied by soaking shoot tips, nodal segments, zygotic embryos, or callus tissues, incorporating them into culture media, or using localized applications such as filter paper. Treatment durations generally range from 6 to 72 h, depending on species‐specific sensitivity. Excessive chemical exposure frequently results in cytotoxicity, mixoploidy, reduced regeneration efficiency, or unstable chimeric tissues. Consequently, successful chromosome doubling is routinely confirmed using flow cytometry, chromosome counting (Schweizer 1973; Sugiura 1936), or stomatal measurements to verify ploidy stability (Dhooghe et al. 2011; Kato et al. 2005; Kuznetsova et al. 2017; Stebbins 1966).

Several comparative studies have demonstrated that the efficiency of chromosome doubling varies considerably among plant species and explant types. For example, oryzalin was reported to be more effective than colchicine for chromosome doubling in isolated cotyledons, whereas colchicine produced higher polyploid induction frequencies in microshoots of *Chaenomeles japonica* (Stanys et al. 2006). These observations highlight the importance of optimizing both the choice of antimitotic agent and the treatment protocol for each experimental system.

The development of chromosome‐doubling methodologies has progressed considerably since the pioneering discovery of colchicine‐induced polyploidy. Advances in plant cytogenetics have substantially improved our understanding of chromosome organization, genome evolution, and meiotic behavior, providing the theoretical foundation for modern chromosome engineering approaches aimed at stabilizing interspecific hybrids and graft‐derived chimeras (Blakeslee and Avery 1937; Stebbins 1966; Grant 1978; Paterson et al. 2000; Kato et al. 2005; Pawlowski 2010; Schubert and Shaw 2011; Schweizer 1973; Sugiura 1936).

Although induced polyploidization has not yet been evaluated in embryonic graft‐derived plants, evidence from plant breeding and wide hybridization studies suggests that chromosome doubling could potentially improve reproductive stability, genome dosage balance, and long‐term physiological performance. When integrated with CRISPR/Cas‐mediated genome editing, chromosome doubling may provide a complementary strategy for stabilizing engineered chimeras by reducing reproductive incompatibility while preserving desirable traits contributed by both graft partners.

**FIGURE 4:**
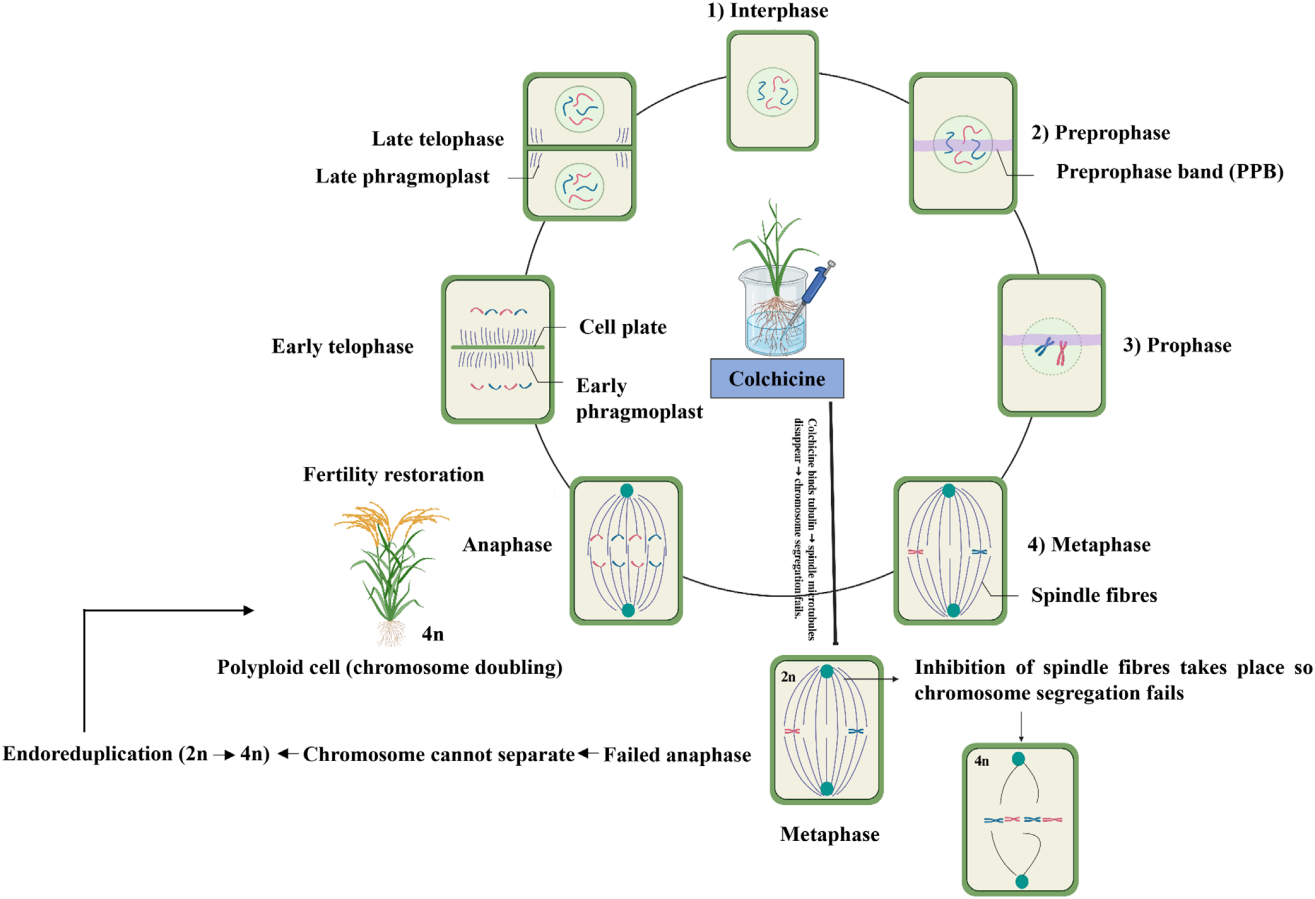
Restoring fertility through chromosome doubling by mitotic spindle inhibition. This figure illustrates the principle of chromosome doubling induced by colchicine‐mediated inhibition of mitotic spindle formation. During normal mitosis, chromosomes progress through interphase, prophase, metaphase, anaphase, and telophase, ensuring accurate chromosome segregation into daughter cells. Colchicine disrupts microtubule spindle formation, preventing chromosome separation during anaphase. Consequently, chromosomes remain within a single nucleus and undergo genome duplication, resulting in chromosome doubling (2n → 4n). Chromosome doubling has been widely used to restore homologous chromosome pairing and improve fertility in sterile or partially sterile hybrids and polyploid breeding systems. The figure is based on established principles of chromosome doubling and polyploid induction in plants (Blakeslee and Avery 1937; Dhooghe et al. 2011; Stanys et al. 2006; Han et al. 2025).

Together, these advances highlight the future potential of integrating embryonic grafting, CRISPR‐guided genome editing, QTL‐assisted compatibility mapping, and induced polyploidization into a unified framework for plant chimera engineering. Although these technologies have not yet been experimentally combined within a single embryonic grafting platform, they represent complementary strategies that could facilitate the future development of reproductively stable, physiologically coordinated, and agriculturally valuable cross‐taxon chimeras, including nitrogen‐fixing cereals and climate‐resilient crop systems (Figure 4).

## 7 Survival, Reproducibility, and Field Transfer: The Bottlenecks

Despite its considerable potential, embryonic grafting continues to face significant biological and technical challenges related to reproducibility, survival, and scalability. Precise alignment of embryonic tissues remains one of the major limitations, particularly in intergeneric and interfamily graft combinations, where differences in embryo size, morphology, and developmental stage complicate the accurate alignment of embryonic polarity and tissue interfaces. Improper alignment may impair vascular reconnection, shoot‐root coordination, and subsequent plant development, ultimately reducing graft success (Reeves et al. 2022; Mukundan et al. 2024).

Successful graft union formation also depends on effective cell adhesion, wound healing, and vascular regeneration. Species‐specific differences in cell wall composition, mechanical properties, hormonal responsiveness, and wound‐healing capacity may reduce graft compatibility by limiting cell‐to‐cell communication, callus organization, and vascular continuity. Although embryonic tissues generally exhibit greater developmental plasticity than mature tissues, the molecular mechanisms governing long‐term compatibility across distantly related taxa remain largely unknown (Melnyk 2017; Feng et al. 2024).

An additional challenge arises during the transition from in vitro culture to ex vitro acclimatization and ultimately to field conditions. Environmental stresses, including desiccation, fluctuating temperature, pathogen exposure, and mechanical stress, frequently reduce survival during acclimatization. Successful transfer therefore requires careful optimization of humidity, light intensity, nutrient availability, and substrate conditions to support physiological adaptation of the developing chimera.

Reproducibility also remains a major limitation because current embryonic grafting protocols are highly species‐dependent. Variations in culture media composition, developmental stage at grafting, growth regulator concentrations, scaffold materials, and environmental conditions can all influence graft success. To date, successful embryonic grafting has been demonstrated in only a limited number of monocot systems, including combinations involving rice, maize, wheat, oat, banana, pineapple, and the recently reported *Zea mays–Dendrobium ovatum* system under carefully controlled laboratory conditions (Reeves et al. 2022; Mukundan et al. 2024). Whether comparable success can be achieved across broader taxonomic groups or under field conditions remains unknown.

Future progress will therefore depend on the development of standardized, reproducible, and species‐flexible embryonic grafting platforms that integrate precise embryo manipulation, optimized tissue culture protocols, advanced imaging technologies, molecular compatibility markers, and automated grafting systems. Such advances will be essential for translating embryonic grafting from a proof‐of‐concept laboratory technique into a scalable platform for crop improvement and synthetic plant engineering.

## 8 Toward Programmable Chimeras and Synthetic Plants

The emergence of embryonic grafting represents a conceptual shift in plant engineering, moving beyond conventional graft propagation toward the rational design of modular plant systems. Rather than focusing solely on overcoming graft incompatibility, future research may enable the development of programmable chimeras, in which rootstock and scion components are selected from taxonomically distant species based on complementary physiological, developmental, or agronomic traits. Such an approach could potentially combine the drought tolerance of one species with the nutritional quality or yield potential of another, or integrate the extensive root architecture of wild relatives with the high productivity of elite crop cultivars.

Advances in developmental biology, synthetic biology, and genome engineering may further expand the capabilities of embryonic grafting. As our understanding of long‐distance signaling, hormonal homeostasis, vascular regeneration, and inter‐organ communication continues to improve, future strategies may incorporate synthetic regulatory circuits, programmable gene expression systems, or CRISPR‐based control modules to regulate growth, resource allocation, stress responses, or developmental timing within graft‐derived chimeras. Although these concepts remain largely theoretical, they provide an exciting framework for designing crop architectures tailored to specific environments and agricultural demands.

Future integration of embryonic grafting with genome editing, synthetic biology, and microbial engineering could also enable increasingly sophisticated plant systems. For example, engineered chimeras may eventually combine complementary traits such as enhanced nutrient acquisition, improved abiotic stress tolerance, optimized photosynthetic performance, or beneficial plant–microbe interactions (Song et al. 2015). While applications such as synthetic cross‐kingdom grafting or the incorporation of entirely novel biological functions remain speculative and have not yet been demonstrated experimentally, continued advances in developmental and molecular engineering may gradually expand the range of compatible biological systems.

Ultimately, embryonic grafting provides a promising conceptual platform for the future development of synthetic plants, where developmental compatibility, physiological integration, and molecular engineering are combined to generate novel crop architectures. Although substantial biological and technical challenges remain, continued progress in embryonic grafting, genome editing, synthetic biology, and systems biology may eventually enable the rational design of programmable plant chimeras with applications in sustainable agriculture, climate resilience, and future crop improvement.

## 9 Discussion

The convergence of embryonic grafting, precision genome editing, and chromosome‐level engineering has considerably expanded the scope of modern grafting biology. Traditional grafting has enabled the transfer of desirable physiological traits between compatible cultivars or closely related species; however, its application remains constrained by taxonomic distance, vascular incompatibility, immune responses, and reproductive barriers (Melnyk 2017; Reeves et al. 2022). These limitations are particularly evident in monocot crops, where the absence of a vascular cambium and distinct developmental programs has historically restricted grafting success.

Recent studies have demonstrated that embryonic grafting provides an alternative developmental window for overcoming some of these anatomical barriers. Reeves et al. (2022) showed that embryonic grafting could successfully establish functional graft unions among several monocot species by exploiting the developmental plasticity of juvenile tissues. Similarly, Mukundan et al. (2024) reported rapid vascular reconnection and high survival rates in embryonic monocot grafts under controlled in vitro conditions. Together, these findings suggest that early embryonic stages provide a favorable environment for graft establishment that differs fundamentally from conventional mature‐tissue grafting.

Although anatomical union represents the first prerequisite for graft success, long‐term physiological integration ultimately depends on coordinated molecular regulation. Graft healing requires synchronized cell‐wall remodeling, hormone signaling, vascular differentiation, oxidative stress regulation, and transcriptional reprogramming (Melnyk 2017; Nanda and Melnyk 2018; Feng et al. 2024). While these mechanisms have been extensively characterized in conventional grafting systems, their operation during embryonic graft development remains largely unexplored. Consequently, molecular pathways identified in compatible grafting systems provide valuable candidate mechanisms for future investigation in embryonic graft‐derived plants.

Similarly, the molecular mechanisms underlying hybrid sterility offer important insights into the long‐term developmental stability of engineered chimeras. Studies in rice have identified several hybrid sterility loci, including *SaF, Sc, OgTPR1, and the ORF3–ORF4–ORF5* complex, together with unfolded protein response (UPR) regulators such as *BiP, bZIP60, and IRE1*, as important determinants of reproductive compatibility and endoplasmic reticulum stress (Rao et al. 2021; Zegeye et al. 2022). Although these pathways have not yet been experimentally validated in embryonic graft‐derived plants, they represent promising molecular candidates for future functional studies aimed at improving reproductive stability across distant taxa.

Rice has emerged as one of the most advanced monocot systems for CRISPR/Cas‐mediated crop improvement. Genome editing has successfully targeted genes controlling hybrid sterility, flowering time, grain development, and abiotic stress adaptation (Zegeye et al. 2022; Zhou et al. 2023). Editing of *TMS5, TMS10, CSA, SaF, OgTPR1, Sc, and ORF2* has contributed to improved fertility management in rice breeding, whereas multiplex editing of *DEP1, GS3, GW2, TGW6, and Gn1a* has enhanced grain yield and architecture (Li et al. 2016; Han et al. 2025). Likewise, editing of *Hd1, Hd3a, RFT1, and Ehd1* has enabled manipulation of flowering behavior under diverse photoperiod conditions (Zegeye et al. 2022). Collectively, these studies establish rice as an important reference system for identifying molecular pathways that may eventually support compatibility engineering in embryonic grafting.

Comparable advances have been achieved in wheat despite the challenges posed by its hexaploid genome. Multiplex CRISPR disruption of Ms1 and its homoeologs (*Ms‐A1 and Ms‐D1*) has enabled efficient production of male‐sterile lines suitable for hybrid seed production without relying on cytoplasmic male sterility systems (Okada et al. 2019). These studies provide valuable molecular resources for reproductive engineering and may guide future investigations into synchronizing reproductive development in embryonic graft‐derived plants.

Dicot crops have likewise contributed important developmental insights. In tomato, CRISPR‐mediated modification of genes regulating meristem identity, vascular regeneration, flowering, fruit ripening, and stress responses, including *WUS, CLV3, SlMADS3, SlAGL6, SlSEP4, RIN, PSY1, HY5, SlMLO1, and SlDMR6*, has substantially advanced understanding of developmental regulation (Thomas et al. 2022; Berman et al. 2025). Similarly, genome editing in potato has demonstrated successful manipulation of starch metabolism, enzymatic browning, disease resistance, and self‐incompatibility through genes such as *GBSSI, PPO2, eIF4E1, StDMR6, and* S‐RNase (Tiwari et al. 2022; Chincinska et al. 2023). Although these studies were not performed in embryonic grafts, they provide valuable molecular frameworks that may inform future engineering of developmental coordination and physiological stability in cross‐taxon chimeras.

In parallel with genome editing, induced chromosome doubling has emerged as an important complementary strategy for improving genomic stability. Treatment with antimitotic agents such as colchicine, oryzalin, trifluralin, and amiprophos‐methyl (APM) has been widely used to restore meiotic stability, fertility, and chromosome pairing in wide hybrids and polyploid crops (Dhooghe et al. 2011). These approaches may complement future embryonic graft engineering by helping stabilize genome dosage and reproductive performance in graft‐derived chimeric plants.

Despite these considerable advances, several important challenges remain before embryonic grafting can be translated into routine crop improvement. Standardized protocols for embryonic explant preparation, graft alignment, sealing strategies, vascular assessment, physiological characterization, and long‐term field evaluation are still lacking. Moreover, integrating transcriptomics, proteomics, metabolomics, hormone profiling, and spatial imaging will be essential for understanding the complex developmental dialogue occurring at the embryonic graft junction.

Overall, embryonic grafting, precision genome editing, chromosome engineering, and molecular breeding collectively represent complementary technologies that may substantially expand the future possibilities of cross‐taxon plant engineering. Continued integration of developmental biology, molecular genetics, and systems biology will determine how effectively these approaches can be translated into stable, reproducible, and agriculturally valuable chimeric crops.

## 10 Concluding Remarks and Future Perspectives

Embryonic grafting represents an emerging frontier at the intersection of developmental biology, plant regeneration, and precision genome engineering. By exploiting the remarkable plasticity of early embryonic tissues, this approach offers opportunities to establish functional associations between taxonomically distant species that remain largely inaccessible through conventional grafting or classical hybridization (Reeves et al. 2022; Mukundan et al. 2024).

Recent advances in CRISPR/Cas technologies have substantially expanded our understanding of the molecular pathways governing fertility, developmental coordination, vascular regeneration, and stress adaptation. Functional characterization of genes regulating hybrid sterility (*ORF3, ORF4, ORF5, SaF*), flowering (*Hd1, Hd3a, Ehd1, FT1*), floral identity (*OsMADS3, OsMADS58, SlMADS3, SlAGL6*), meristem maintenance (*WUS, CLV3*), and vascular regeneration provides a valuable molecular foundation for future optimization of embryonic graft‐derived systems (Rao et al. 2021; Zegeye et al. 2022; Zhou et al. 2023; Thomas et al. 2022).

Similarly, chromosome doubling through induced polyploidization has demonstrated considerable potential for restoring fertility and genomic balance in wide hybrids (Dhooghe et al., 2011). Although the application of this strategy to plants generated through embryonic grafting remains hypothetical, integrating chromosome engineering with genome editing represents an attractive direction for future research aimed at improving developmental stability and long‐term reproductive performance.

From a broader perspective, embryonic grafting offers several conceptual advantages over conventional transgenic approaches. Because each graft partner retains its native genome, this strategy may circumvent some of the biological limitations associated with wide hybridization while preserving opportunities for physiological integration between genetically distinct species. However, its practical implementation still requires extensive experimental validation before it can be considered a reliable platform for crop improvement.

Several major knowledge gaps remain. The molecular mechanisms governing long‐distance signaling, small RNA movement, epigenetic reprogramming, vascular maturation, hormonal coordination, and reproductive synchronization across taxonomic boundaries remain poorly understood. Addressing these questions through integrated multi‐omics analyses, advanced imaging technologies, and functional genome editing will be essential for establishing predictive models of graft compatibility (Melnyk 2017; Feng et al. 2024; Augstein and Melnyk 2025).

Ultimately, embryonic grafting should be regarded as a promising developmental technology whose future success will depend on combining developmental biology, molecular genetics, cytogenetics, and precision genome engineering. Continued advances in these complementary disciplines may eventually enable the rational design of stable, fertile, and physiologically integrated cross‐taxon graft plants and, where cellular integration is achieved, engineered chimeras, thereby expanding the possibilities for future crop improvement and sustainable agriculture.

## Funding

The authors have nothing to report.

## Ethics Statement

The authors have nothing to report.

## Consent

The authors have nothing to report.

## Conflicts of Interest

The authors declare no conflicts of interest.

## Data Availability

Data sharing not applicable to this article as no datasets were generated or analysed during the current study.

## References

[pei370208-bib-0001] Augstein, F. , and C. W. Melnyk . 2025. “Modern and Historical Uses of Plant Grafting to Engineer Development, Stress Tolerance, Chimeras, and Hybrids.” Plant Journal 121, no. 4: e70057. 10.1111/tpj.70057 PMC11844807 39982814

[pei370208-bib-0002] Belmonte‐Ureña, L. J. , J. A. Garrido‐Cardenas , and F. Camacho‐Ferre . 2020. “Analysis of World Research on Grafting in Horticultural Plants.” HortScience 55, no. 1: 112–120.

[pei370208-bib-0003] Berman, A. , N. Su , Z. Li , et al. 2025. “Construction of Multi‐Targeted CRISPR Libraries in Tomato to Overcome Functional Redundancy at Genome‐Scale Level.” Nature Communications 16, no. 1: 4111. 10.1038/s41467-025-59280-6 PMC12048548 40316524

[pei370208-bib-0004] Bhatnagar, S. P. , and P. K. Dantu . 2015. The Embryology of Angiosperms. Vikas Publishing House.

[pei370208-bib-0005] Bhattacharya, K. R. 2011. Rice Quality: A Guide to Rice Properties and Analysis. Elsevier.

[pei370208-bib-0006] Bin Rahman, A. R. , and J. Zhang . 2023. “Trends in Rice Research: 2030 and Beyond.” Food and Energy Security 12, no. 2: e390.

[pei370208-bib-0007] Blakeslee, A. F. , and A. G. Avery . 1937. “Methods of Inducing Doubling of Chromosomes in Plants by Treatment With Colchicine.” Journal of Heredity 28, no. 12: 393–411.

[pei370208-bib-0008] Chincinska, I. A. , M. Miklaszewska , and D. Sołtys‐Kalina . 2023. “Recent Advances and Challenges in Potato Improvement Using CRISPR/Cas Genome Editing.” Planta 257, no. 1: 25. 10.1007/s00425-022-04054-3 PMC9789015 36562862

[pei370208-bib-0009] Colla, G. , Y. Rouphael , M. Cardarelli , D. Massa , A. Salerno , and E. Rea . 2006. “Yield, Fruit Quality and Mineral Composition of Grafted Melon Plants Grown Under Saline Conditions.” Journal of Horticultural Science and Biotechnology 81, no. 1: 146–152.

[pei370208-bib-0010] Colla, G. , Y. Rouphael , M. Cardarelli , and E. Rea . 2006. “Effect of Salinity on Yield, Fruit Quality, Leaf Gas Exchange, and Mineral Composition of Grafted Watermelon Plants.” HortScience 41, no. 3: 622–627.

[pei370208-bib-0011] Colla, G. , C. M. C. Suarez , M. Cardarelli , and Y. Rouphael . 2010. “Improving Nitrogen Use Efficiency in Melon by Grafting.” HortScience 45, no. 4: 559–565. 10.21273/HORTSCI.45.4.559.

[pei370208-bib-0012] Darikova, J. A. , Y. V. Savva , E. A. Vaganov , A. M. Grachev , and G. V. Kuznetsova . 2011. “Grafts of Woody Plants and the Problem of Incompatibility Between Scion and Rootstock.” Journal of Siberian Federal University. Biology 4, no. 1: 54–63.

[pei370208-bib-0013] Dhooghe, E. , K. Van Laere , T. Eeckhaut , L. Leus , and J. Van Huylenbroeck . 2011. “Mitotic Chromosome Doubling of Plant Tissues In Vitro.” Plant Cell, Tissue and Organ Culture 104, no. 3: 359–373. 10.1007/s11240-010-9786-5.

[pei370208-bib-0014] Edelstein, M. , Z. Plaut , and M. Ben‐Hur . 2011. “Sodium and Chloride Exclusion and Retention by Non‐Grafted and Grafted Melon and *Cucurbita* Plants.” Journal of Experimental Botany 62, no. 1: 177–184. 10.1093/jxb/erq255 PMC2993908 20729482

[pei370208-bib-0016] Enciso‐Rodriguez, F. , N. C. Manrique‐Carpintero , S. S. Nadakuduti , C. R. Buell , D. Zarka , and D. Douches . 2019. “Overcoming Self‐Incompatibility in Diploid Potato Using CRISPR‐Cas9.” Frontiers in Plant Science 10: 376. 10.3389/fpls.2019.00376 PMC6454193 31001300

[pei370208-bib-0017] Feng, M. , F. Augstein , A. Kareem , and C. W. Melnyk . 2024. “Plant Grafting: Molecular Mechanisms and Applications.” Molecular Plant 17, no. 1: 75–91. 10.1016/j.molp.2023.12.006 38102831

[pei370208-bib-0018] Fernández‐García, N. , V. Martínez , A. Cerdá , and M. Carvajal . 2002. “Water and Nutrient Uptake of Grafted Tomato Plants Grown Under Saline Conditions.” Journal of Plant Physiology 159, no. 8: 899–905. 10.1078/0176-1617-00809.

[pei370208-bib-0019] Gohil, S. , A. Kumari , A. Prakash , N. Shah , S. Bhutani , and M. Singh . 2024. “CRISPR‐Based Industrial Crop Improvements.” In Industrial Crop Plants, edited by M. Singh , 123–162. Springer. 10.1007/978-981-97-1003-4_5.

[pei370208-bib-0020] Grant, W. F. 1978. “Chromosome Aberrations in Plants as a Monitoring System.” Environmental Health Perspectives 27: 37–43. 10.1289/ehp.782737. PMC1637299 367773

[pei370208-bib-0021] Han, X. , J. Li , G. Li , et al. 2025. “Rapid Formation of Stable Autotetraploid Rice From Genome‐Doubled F1 Hybrids of *Japonica–Indica* Subspecies.” Nature Plants 11: 1–18. 10.1038/s41477-025-01966-2. PMC12015120 40164786

[pei370208-bib-0022] Howell, S. H. 2013. “Endoplasmic Reticulum Stress Responses in Plants.” Annual Review of Plant Biology 64: 477–499. 10.1146/annurev-arplant-050312-120053. 23330794

[pei370208-bib-0023] Jin, S. B. , D. H. Lee , S. M. Park , J. S. Park , and Y. E. Moon . 2025. “Intergeneric Plants Developed by Protoplast Fusion Between Two *Citrus* Hybrids for Producing High‐Quality Fruit.” Scientia Horticulturae 343: 112345. 10.1016/j.scienta.2025.112345.

[pei370208-bib-0024] Kato, A. , J. M. Vega , F. Han , J. C. Lamb , and J. A. Birchler . 2005. “Advances in Plant Chromosome Identification and Cytogenetic Techniques.” Current Opinion in Plant Biology 8, no. 2: 148–154. 10.1016/j.pbi.2005.01.014. 15752994

[pei370208-bib-0025] Kawaguchi, M. , A. Taji , D. Backhouse , and M. Oda . 2008. “Anatomy and Physiology of Graft Incompatibility in Solanaceous Plants.” Journal of Horticultural Science and Biotechnology 83, no. 5: 581–588. 10.1080/14620316.2008.11512427.

[pei370208-bib-0026] Kragler, F. , and R. Bock . 2025. “The Biology of Grafting and Its Applications in Studying Information Exchange Between Plants.” Nature Plants 11, no. 5: 955–966. 10.1038/s41477-025-01982-2. 40200023

[pei370208-bib-0027] Kumari, M. , L. K. Dashora , M. Jain , R. Meena , and Y. Tak . 2023. “Effect of Auxin and Cytokinin on Grafting and Growth of Sapling of Guava ( *Psidium guajava* L.).” International journal of environment and climate change 13, no. 9: 1–8.

[pei370208-bib-0028] Kuznetsova, M. A. , I. A. Chaban , and E. V. Sheval . 2017. “Visualization of Chromosome Condensation in Plants With Large Chromosomes.” BMC Plant Biology 17, no. 1: 153. 10.1186/s12870-017-1093-3. PMC5596468 28899358

[pei370208-bib-0029] Lee, J. M. 1994. “Cultivation of Grafted Vegetables I. Current Status, Grafting Methods, and Benefits.” HortScience 29, no. 4: 235–239.

[pei370208-bib-0030] Lewis, W. J. , and D. Alexander . 2008. Grafting and Budding: A Practical Guide for Fruit and Nut Plants and Ornamentals. Landlinks Press.

[pei370208-bib-0031] Li, M. , X. Li , Z. Zhou , et al. 2016. “Reassessment of the Four Yield‐Related Genes *Gn1a*, *DEP1*, *GS3*, and *IPA1* in Rice Using a CRISPR/Cas9 System.” Frontiers in Plant Science 7: 377. 10.3389/fpls.2016.00377 PMC4811884 27066031

[pei370208-bib-0032] Li, Y. , W. Sun , F. Liu , et al. 2019. “Methods for Grafting *Arabidopsis thaliana* and *Eutrema salsugineum* .” Plant Methods 15, no. 1: 93. 10.1186/s13007-019-0477-x. PMC6691545 31417609

[pei370208-bib-0033] Li, Y. , and D. Zhao . 2021. “Transcriptome Analysis of Scions Grafted to Potato Rootstock for Improving Late Blight Resistance.” BMC Plant Biology 21, no. 1: 272. 10.1186/s12870-021-03039-w. PMC8204497 34130637

[pei370208-bib-0034] Melnyk, C. W. 2017. “Plant Grafting: Insights Into Tissue Regeneration.” Regeneration 4: 3–14. 10.1002/reg2.71. PMC5350079 28316790

[pei370208-bib-0036] Moore, R. 1984. “A Model for Graft Compatibility–Incompatibility in Higher Plants.” American Journal of Botany 71, no. 5: 752–758. 10.1002/j.1537-2197.1984.tb14182.x.

[pei370208-bib-0037] Mudge, K. , J. Janick , S. Scofield , and E. E. Goldschmidt . 2009. “A History of Grafting.” Horticultural Reviews 35: 437–493.

[pei370208-bib-0038] Mujtaba, M. , D. Wang , L. B. Carvalho , et al. 2021. “Nanocarrier‐Mediated Delivery of miRNA, RNAi, and CRISPR‐Cas for Plant Protection: Current Trends and Future Directions.” ACS Agricultural Science & Technology 1, no. 5: 417–435.

[pei370208-bib-0039] Mukundan, N. S. , A. Patil , D. S. Mense , S. L. Rodrigues , and V. S. Babu . 2024. “Bridging Photosynthetic Worlds: Creating C4‐CAM Chimeras Through Embryo Grafting in Maize and Orchids.” Scientia Horticulturae 336: 112345. 10.1016/j.scienta.2024.112345.

[pei370208-bib-0040] Mushtaq, M. , J. A. Bhat , Z. A. Mir , et al. 2018. “CRISPR/Cas Approach: A New Way of Looking at Plant‐Abiotic Interactions.” Journal of Plant Physiology 224: 156–162. 10.1016/j.jplph.2018.04.001 29655033

[pei370208-bib-0041] Muzik, T. J. , and C. D. La Rue . 1954. “Further Studies on the Grafting of Monocotyledonous Plants.” American Journal of Botany 41: 448–455. 10.2307/2438855.

[pei370208-bib-0042] Nanda, A. K. , and C. W. Melnyk . 2018. “The Role of Plant Hormones During Grafting.” Journal of Plant Research 131, no. 1: 49–58. 10.1007/s10265-017-0994-5. PMC5762790 29181647

[pei370208-bib-0043] Navarro, L. , C. N. Roistacher , and T. Murashige . 1975. “Improvement of Shoot‐Tip Grafting In Vitro for Virus‐Free Citrus.” Journal of the American Society for Horticultural Science 100, no. 5: 471–479. 10.21273/JASHS.100.5.471.

[pei370208-bib-0044] Nawaz, M. A. , M. Imtiaz , Q. Kong , et al. 2016. “Grafting: A Technique to Modify Ion Accumulation in Horticultural Crops.” Frontiers in Plant Science 7: 1457. 10.3389/fpls.2016.01457. PMC5073839 27818663

[pei370208-bib-0045] Notaguchi, M. , K. I. Kurotani , Y. Sato , et al. 2020. “Cell‐Cell Adhesion in Plant Grafting Is Facilitated by β‐1,4‐Glucanases.” Science 369, no. 6504: 698–702. 10.1126/science.abc3710 32764072

[pei370208-bib-0046] Okada, A. , T. Arndell , N. Borisjuk , et al. 2019. “CRISPR/Cas9‐Mediated Knockout of *Ms1* Enables the Rapid Generation of Male‐Sterile Hexaploid Wheat Lines for Use in Hybrid Seed Production.” Plant Biotechnology Journal 17, no. 10: 1905–1913. 10.1111/pbi.13106. PMC6737020 30839150

[pei370208-bib-0047] Paterson, A. H. , J. E. Bowers , M. D. Burow , et al. 2000. “Comparative Genomics of Plant Chromosomes.” Plant Cell 12, no. 9: 1523–1539. 10.1105/tpc.12.9.1523. PMC149067 11006329

[pei370208-bib-0048] Pawlowski, W. P. 2010. “Chromosome Organization and Dynamics in Plants.” Current Opinion in Plant Biology 13, no. 6: 640–645. 10.1016/j.pbi.2010.09.012. 20970369

[pei370208-bib-0049] Pérez‐Luna, A. L. , C. Wehenkel , J. A. Prieto‐Ruíz , et al. 2020. “Grafting in Conifers: A Review.” Pakistan Journal of Botany 52, no. 4: 1369–1378. 10.30848/PJB2020-4(36).

[pei370208-bib-0050] Rao, J. , X. Wang , Z. Cai , Y. Fan , and J. Yang . 2021. “Genetic Analysis of S5‐Interacting Genes Regulating Hybrid Sterility in Rice.” Rice 14, no. 1: 11. 10.1186/s12284-020-00452-x. PMC7797014 33423160

[pei370208-bib-0051] Rasool, A. , S. Mansoor , K. M. Bhat , et al. 2020. “Mechanisms Underlying Graft Union Formation and Rootstock–Scion Interaction in Horticultural Plants.” Frontiers in Plant Science 11: 590847. 10.3389/fpls.2020.590847. PMC7758432 33362818

[pei370208-bib-0052] Reeves, G. , A. Tripathi , P. Singh , et al. 2022. “Monocotyledonous Plants Graft at the Embryonic Root–Shoot Interface.” Nature 602: 280–286. 10.1038/s41586-022-04344-0. 34937943

[pei370208-bib-0053] Rivero, R. M. , J. M. Ruiz , and L. Romero . 2003a. “Can Grafting in Tomato Plants Strengthen Resistance to Thermal Stress?” Journal of the Science of Food and Agriculture 83, no. 13: 1315–1319. 10.1002/jsfa.1541.

[pei370208-bib-0054] Rivero, R. M. , J. M. Ruiz , and L. Romero . 2003b. “Role of Grafting in Horticultural Plants Under Stress Conditions.” Journal of Food, Agriculture & Environment 1: 70–74.

[pei370208-bib-0055] Roy, D. , P. Mehra , L. Clark , et al. 2025. “Redox‐Regulated Aux/IAA Multimerization Modulates Auxin Responses.” Science 389: eadu1470. 10.1126/science.adu1470. 40504951

[pei370208-bib-0056] Rubab, U. E. , S. Hussain , A. Ashraf , M. Saeed , N. I. Raja , and Z. U. R. Mashwani . 2025. “Revolutionizing Agricultural Innovation: Game‐Changing Nanotechnology for the Future of Vegetable and Fruit Production.” BioNanoScience 15, no. 4: 562. 10.1007/s12668-025-02104-0.

[pei370208-bib-0057] Schubert, I. , and P. Shaw . 2011. “Organization and Dynamics of Plant Interphase Chromosomes.” Trends in Plant Science 16, no. 5: 273–281. 10.1016/j.tplants.2011.02.002. 21393049

[pei370208-bib-0058] Schweizer, D. 1973. “Differential Staining of Plant Chromosomes With Giemsa.” Chromosoma 40, no. 3: 307–320. 10.1007/BF00326184.

[pei370208-bib-0059] Singh, H. , P. Kumar , A. Kumar , M. C. Kyriacou , G. Colla , and Y. Rouphael . 2020. “Grafting Tomato as a Tool to Improve Salt Tolerance.” Agronomy 10, no. 2: 263. 10.3390/agronomy10020263.

[pei370208-bib-0060] Song, G. Q. , A. E. Walworth , and W. H. Loescher . 2015. “Grafting of Genetically Engineered Plants.” Journal of the American Society for Horticultural Science 140, no. 3: 203–213. 10.21273/JASHS.140.3.203.

[pei370208-bib-0061] Stanys, V. , A. Weckman , G. Staniene , and P. Duchovskis . 2006. “In Vitro Induction of Polyploidy in Japanese Quince ( *Chaenomeles japonica* ).” Plant Cell, Tissue and Organ Culture 84, no. 3: 263–268.

[pei370208-bib-0062] Stebbins, G. L. 1966. “Chromosomal Variation and Evolution: Polyploidy and Chromosome Size and Number Shed Light on Evolutionary Processes in Higher Plants.” Science 152, no. 3728: 1463–1469. 10.1126/science.152.3728.1463. 17788022

[pei370208-bib-0063] Sugiura, T. 1936. “Studies on the Chromosome Numbers in Higher Plants, With Special Reference to Cytokinesis I.” Cytologia 7, no. 4: 544–595. 10.1508/cytologia.7.544.

[pei370208-bib-0064] Thomas, H. , L. Van den Broeck , R. Spurney , R. Sozzani , and M. Frank . 2022. “Gene Regulatory Networks for Compatible Versus Incompatible Grafts Identify a Role for *SlWOX4* During Junction Formation.” Plant Cell 34, no. 1: 535–556. 10.1093/plcell/koab246. PMC8846177 34609518

[pei370208-bib-0065] Tiwari, J. K. , T. Buckseth , C. Challam , et al. 2022. “CRISPR/Cas Genome Editing in Potato: Current Status and Future Perspectives.” Frontiers in Genetics 13: 827808. 10.3389/fgene.2022.827808. PMC8849127 35186041

[pei370208-bib-0066] Tiwari, J. K. , A. K. Singh , and T. K. Behera . 2023. “CRISPR/Cas Genome Editing in Tomato Improvement: Advances and Applications.” Frontiers in Plant Science 14: 1121209. 10.3389/fpls.2023.1121209. PMC9995852 36909403

[pei370208-bib-0067] Tsutsui, H. , and M. Notaguchi . 2017. “The Use of Grafting to Study Systemic Signaling in Plants.” Plant and Cell Physiology 58, no. 8: 1291–1301. 10.1093/pcp/pcx098. 28961994

[pei370208-bib-0068] Turnbull, C. G. , J. P. Booker , and H. M. O. Leyser . 2002. “Micrografting Techniques for Testing Long‐Distance Signalling in *Arabidopsis* .” Plant Journal 32, no. 2: 255–262. 10.1046/j.1365-313X.2002.01419.x. 12383090

[pei370208-bib-0069] Vanneste, S. , Y. Pei , and J. Friml . 2025. “Mechanisms of Auxin Action in Plant Growth and Development.” Nature Reviews Molecular Cell Biology 26: 648–666. 10.1038/s41580-025-00851-2. 40389696

[pei370208-bib-0070] Wang, J. , L. Jiang , and R. Wu . 2017. “Plant Grafting: How Genetic Exchange Promotes Vascular Reconnection.” New Phytologist 214, no. 1: 56–65. 10.1111/nph.14383. 27991666

[pei370208-bib-0071] Wang, M. , Z. Li , P. R. Matthews , N. M. Upadhyaya , and P. M. Waterhouse . 1997. “Improved Vectors for *Agrobacterium tumefaciens* ‐Mediated Transformation of Monocot Plants.” Acta Horticulturae 461: 401–408.

[pei370208-bib-0072] Yang, L. , L. Xia , Y. Zeng , Q. Han , and S. Zhang . 2022. “Grafting Enhances Plant Drought Resistance: Current Understanding, Mechanisms, and Future Perspectives.” Frontiers in Plant Science 13: 1015317. 10.3389/fpls.2022.1015317. PMC9583147 36275555

[pei370208-bib-0073] Zegeye, W. A. , M. Tsegaw , Y. Zhang , and L. Cao . 2022. “CRISPR‐Based Genome Editing: Advancements and Opportunities for Rice Improvement.” International Journal of Molecular Sciences 23, no. 8: 4454. 10.3390/ijms23084454. PMC9027422 35457271

[pei370208-bib-0074] Zhang, A. , T. Wang , L. Yuan , et al. 2024. “Horizontal Transfer of Plasmid‐Like Extrachromosomal Circular DNAs Across Graft Junctions in Solanaceae.” Molecular Horticulture 4, no. 1: 41. 10.1186/s43897-024-00124-0. PMC11577957 39563413

[pei370208-bib-0075] Zhang, G. , and H. Guo . 2019. “Effects of Tomato and Potato Heterografting on Photosynthesis, Quality and Yield of Grafted Parents.” Horticulture, Environment, and Biotechnology 60, no. 1: 9–18. 10.1007/s13580-018-0104-5.

[pei370208-bib-0076] Zhang, T. , Y. Zhao , J. Ye , et al. 2019. “Establishing CRISPR/Cas13a Immune System Conferring RNA Virus Resistance in Both Dicot and Monocot Plants.” Plant Biotechnology Journal 17, no. 7: 1185–1187. 10.1111/pbi.13095. PMC6576088 30785668

[pei370208-bib-0077] Zhou, X. , Y. Zhao , P. Ni , Z. Ni , Q. Sun , and Y. Zong . 2023. “CRISPR‐Mediated Acceleration of Wheat Improvement: Advances and Perspectives.” Journal of Genetics and Genomics 50, no. 11: 815–834. 10.1016/j.jgg.2023.09.007. 37741566

